# Demographic Change Across the Lifespan of Pet Dogs and Their Impact on Health Status

**DOI:** 10.3389/fvets.2018.00200

**Published:** 2018-08-23

**Authors:** Lisa J. Wallis, Dóra Szabó, Boglárka Erdélyi-Belle, Enikö Kubinyi

**Affiliations:** Department of Ethology, Eötvös Loránd University, Budapest, Hungary

**Keywords:** aging, lifespan, healthspan, dog-human bond, obesity, trauma, welfare, dog

## Abstract

Although dogs' life expectancies are six to twelve times shorter than that of humans, the demographics (e. g., living conditions) of dogs can still change considerably with aging, similarly to humans. Despite the fact that the dog is a particularly good model for human healthspan, and the number of aged dogs in the population is growing in parallel with aged humans, there has been few previous attempts to describe demographic changes statistically. We utilized an on-line questionnaire to examine the link between the age and health of the dog, and owner and dog demographics in a cross-sectional Hungarian sample. Results from univariate analyses revealed that 20 of the 27 demographic variables measured differed significantly between six dog age groups. Our results revealed that pure breed dogs suffered from health problems at a younger age, and may die at an earlier age than mixed breeds. The oldest dog group (>12 years) consisted of fewer pure breeds than mixed breeds and the mixed breeds sample was on average older than the pure breed sample. Old dogs were classified more frequently as unhealthy, less often had a “normal” body condition score, and more often received medication and supplements. They were also more often male, neutered, suffered health problems (such as sensory, joint, and/or tooth problems), received less activity/interaction/training with the owner, and were more likely to have experienced one or more traumatic events. Surprisingly, the youngest age group contained more pure breeds, were more often fed raw meat, and had owners aged under 29 years, reflecting new trends among younger owners. The high prevalence of dogs that had experienced one or more traumatic events in their lifetime (over 40% of the sample), indicates that welfare and health could be improved by informing owners of the greatest risk factors of trauma, and providing interventions to reduce their impact. Experiencing multiple life events such as spending time in a shelter, changing owners, traumatic injury/prolonged disease/surgery, getting lost, and changes in family structure increased the likelihood that owners reported that their dogs currently show behavioral signs that they attribute to the previous trauma.

## Introduction

A quarter of all households in the UK own a dog, and this figure rises to 33% in Hungary (1) and 44% in the USA (2, 3). Most pet dogs live in close proximity to their owners, sharing living spaces in the home and public outdoor spaces, and some even provide emotional, physical and health related benefits (4). More and more dog owners are viewing their dogs as family members, which has resulted in increased expenditure on dog-related products, and even significant lifestyle changes for dog owners (5). However, 10 years after the implementation of the Animal Welfare Act (2006), according to the PDSA's animal wellbeing report (6), thirty nine percent of owners surveyed stated they were familiar with the act, a decline from 45% in 2011. Worryingly, owners who did not feel informed about each of the five welfare needs were more likely to underestimate the lifetime cost of their pet, and as expense is given as the primary reason for not providing preventative care, knowledge of the cost of ownership is important to ensure that dogs' welfare needs are met. In addition, 25% of dogs surveyed had not received their first initial round of vaccinations. Many owners feed inappropriate foods such as table scraps as part of their dogs main meal, most do not consider their dog's life stage when selecting a diet, and some are not able to recognize when their pets are overweight or obese (7, 8). Dogs go through similar stages of development as humans including—puppyhood (termed childhood in humans), adolescence, adult-hood (starts between 1 and 3 years of age), the senior years (begins between 6 and 10 years of age), and a geriatric phase (7–11 years) (9). In addition, dogs' nutritional requirements change as they age and depend on their activity levels in the same way as humans do. Therefore, it may come as no surprise that up to 60% of dogs in the UK are now classified as overweight or obese, a rise of around 20% since 2007 (6, 8, 10), mirroring the rise in obesity in humans (11). Obesity leads to a reduction in quality of life, shortened longevity and an increase in health issues (12). The evidence presented suggests that dog owners still need to be informed about the various aspects of dog keeping, particularly of the importance of understanding and managing their dog's needs, which change as the dog ages.

Previous studies have identified both physiological declines and changes in dog demographics with age, such as an increase in the occurrence of mobility, sensory and health problems, medicine use, and changes in body condition score (13). Aging in dogs is also associated with a decline in perceptual and cognitive functions (14–19). These declines may result in problematic behavioral changes, ranging from increased vocalization, aggression and phobias, to a loss in house training, which may affect quality of life of the individual and the human-animal bond (16, 20). Whilst it is clear that as age increases, the prevalence of age-related diseases will also increase, there is still some controversy over what is the normal rate of physical and cognitive decline experienced in healthy dogs. Even “successful” aging results in some decline in sensorimotor control, cognitive abilities and behavioral changes. However, the rate of deterioration should not affect the individual's day-to-day functioning; otherwise, this might indicate a pathological problem (16). Despite the growing number of aged dogs in the population, very little is known about the actual prevalence and risk factors of age-related changes in dogs (21).

Many owners are able to detect age-rated physiological and behavioral changes in their dogs and make changes in their daily routine in order to adapt to their dogs current needs (e.g., as dogs' activity levels decrease, owners may take them out for walks less, and participate in fewer training activities). However, the situation is complicated by the fact that the transition from adult to senior or geriatric life stage and the classification of body condition score varies between individuals and their measure is entirely subjective. The terms “aging” and “old,” “senior” and “geriatric,” “overweight,” and “obese” may mean different things to different dog owners (22). Therefore, it is also possible that there are individual biases in owner's opinions on what the “normal” aging process entails, and what a “normal” weight should look like, such that some owners are likely to make changes in their dog keeping/lifestyle practices even if the dog shows no clear signs of aging or disease. Larsen and Farcas (22) have suggested that all senior dogs should be assessed by their veterinarian in order to facilitate detection of changes to their physical, lifestyle and nutritional needs.

In humans, genetic, environmental and social factors, such as gender, previous trauma, stress, lifestyle including diet and exercise, education, occupation, and economic circumstances have been found to influence health and lifespan, and thus may also have an impact on our canine companions (23–25). There is a huge variation in mean life span across the different breeds of dogs living in human households, varying from 7 to 14 (26). An individual's rate of aging is related to its genetic makeup and is influenced by the environment and past experiences; therefore, the age at which senescence begins is likely to differ depending on breed, size and weight (the larger and heavier the breed the lower the age of onset) (27), as well as the prevalence of hereditary diseases (24). Body weight was found to be more predictive of lifespan than either height, breed or breed group (28), and it explained about 44% of the variance in mortality risk amongst 74 dog breeds after the onset of senescence (27). These findings lead to the assumption that large heavy dogs age at a faster rate than smaller dogs (29). Kraus et al. (27) determined that although the age at which mortality started increasing did not differ across small and large breeds, once senescence begins, big dog breeds do age more rapidly than small ones. However, Salvin et al. (17) found little evidence for an increased rate of behavioral aging in large, short-lived dogs utilizing a cross-sectional survey, perhaps due to the shorter window of senescence onset and mortality in large breed dogs.

The term “healthspan” has been attributed to the period of time during which humans and non-human animals are generally healthy and free from serious or chronic illness. The dog is a particularly good model species to examine healthspan, as like in humans, dogs generally have shorter healthspans than lifespans, they are subjected to the same environmental factors, and they develop the same age-related changes and diseases of aging (30, 31). As stated previously, body weight accounts for much of the variation in the timing of death across dog breeds; body weight also influences the age of onset of many age-related diseases such as cancer (32, 33). However, one study found that body weight had no significant influence on health status (as measured by the total number of morbidities) (34). Additionally, mixed breed dogs are often assumed to have a phenotypic advantage over pure breeds, resulting in greater longevity, improved health and lower susceptibility to diseases due to higher genetic variation (26, 29, 35–37). However, Salvin et al. (17) found no evidence for differences between pure breed and cross breed dogs in behavioral aging, and health status is dependent almost exclusively on age with no detectable effect of breed (34). But specific types of morbidities have been found to be breed specific, such as mast cell tumor, lymphoma, granulomatous colitis, and idiopathic epilepsy (33, 38).

Sex differences in longevity and healthspan in dogs depends critically on neuter status. Exceptional longevity is accompanied by a significant delay in the onset of major life-threatening diseases (39). Dogs that are sterilized generally have a longer lifespan than reproductively intact dogs (40), but tend to have different causes of death. Intact dogs are at greater risk for infectious and traumatic causes of death and sterilized dogs have an increased risk for neoplastic and immune-mediated causes of death. Another study reported that in reproductively intact dogs, male dogs lived slightly longer than females, but among sterilized dogs, females live longer than males (41). However, the effect of neutering was greater than the effect of sex.

As discussed, biological variables (such as sex, neutering, body weight, and age) can contribute to the development of chronic disease in humans and dogs, however, other non-biological factors such as environmental, behavioral, social, and economic factors may also have profound effects on canine and human health. Results from behavioral aging, longevity and health status surveys could be confounded by differences in dog and owner demographics, between the different age groups and/or breed groups. For example, Turcsán et al. (42) found that twelve out of 20 demographic and dog keeping factors differed between purebred and mixed-breed dogs, and when they controlled for these differences, some of the previously found associations between the demographic and environmental factors and the behavioral traits measured changed. Therefore, in some cases, differences in these factors resulted in behavior differences between mixed-breeds and purebreds. Other studies have also emphasized the importance of taking into account dog and owner characteristics when examining behavioral traits in dogs (43–45). All point to the fact that an extensive examination into the differences in demographic and environmental factors between different dog age groups would be highly desirable, and could help to emphasize which factors are particularly relevant for aging research, and should be included in subsequent studies on aging related changes in behavior, cognition, longevity and healthspan.

The influence of environmental factors on aging and healthspan remains poorly understood, apart from the obvious culprits, smoking and obesity. Recent research has demonstrated that dogs living in smoking homes are more likely to suffer from DNA damage and show signs of premature aging than those living in non-smoking homes (46). Previous studies have estimated that between 20 and 40% of the pet dog population are classified as obese, and these dogs have elevated levels of inflammatory markers (TNF-alpha and C-reactive protein) (47). Obesity can have detrimental effects on health and longevity; dogs which are overweight are at risk of developing other diseases such as diabetes mellitus, osteoarthritis and urinary incontinence, as well as altered respiratory function (12), and as such obesity is now considered the biggest health and welfare issue affecting pet dogs today. Lifestyle and dietary factors, breed predispositions, underlying diseases, neutering, and aging all may contribute to the development of obesity in dogs (12, 48).

There is evidence that chronic stress can have negative effects on health and lifespan in the domestic dog (49). Previous studies utilizing owner questionnaires have found that the environment, in which the dog is kept, as well as the management choices of the owner (such as how much time they spend with the dog), can vary significantly with the age of the dog, and can also influence healthspan and wellbeing. For example, Bennett and Rohlf (50), established that the owner's perception of their dog's behavior is related to the degree to which the dog is included in its owner's activities, and suggested that the dog–owner relationship may be mediated by participation in shared activities such as hugging, taking the dog in the car, grooming, buying/giving treats, and playing games. As dog age increased, a decrease in shared activities was found, which resulted in reductions in the quality of the dog–owner relationship. Utilizing a different questionnaire, Marinelli et al. (51) found dog age and length of the dog-owner relationship negatively influenced quality of life, physical condition and care of the dog. Older dogs received less medical assistance, which may indicate a failure in the dog–owner relationship, and/or that owners are not well informed about geriatric dog care.

Other than the research examining risk factors for obesity, so far there have been very few studies examining what factors influence health status in pet dogs. Enhanced understanding of the influence of demographic factors on dog health could help to improve welfare in domestic dogs. The aim of this study was to investigate the relationship of dog age on dog and owner demographics in a cross-sectional sample, and additionally, to identify the key variables associated with health status in dogs. Since this study was exploratory, we included a total of 27 dog and owner demographic factors in our analysis. From previous studies, age group, body condition score, and weight are all factors that have been found to influence health, such that older dogs and dogs classified as overweight/obese are more likely to suffer from health problems.

## Methods

### Ethical statement

Data were collected from Hungarian dog owners via an online questionnaire. Owners gave their informed consent for the data to be used for scientific purposes in an introductory letter, before filling out the questionnaire voluntarily and anonymously. A copy of the questionnaire translated into English is available in the Supplementary Materials.

### Subjects

Hungarian dog owners were invited to fill out an online questionnaire, which was advertised on the Eötvös Loránd University Department of Ethology's homepage (http://kutyaetologia.elte.hu), and on the Facebook group “Családi Kutya Program.” The questionnaire was available from the middle of May to the beginning of July 2016. Dogs aged under 1 year were excluded as previous research has suggested that their behavior does not remain stable over time (52). Duplicate entries and entries with missing information were deleted, which resulted in data from a total of 1,207 individual dogs. The full sample consisted of 66% pure breeds, 54% females, of which 17% were intact, and 37% were neutered (26% intact males and 20% neutered males). The descriptive statistics of the sample are presented in Tables 1, 2. Based on the data of the Hungarian Veterinary Chamber (53), in Hungary there are 2 million dogs, of which more than 60% are purebreds. Please refer to the Supplementary Material for a list of the dog breeds in the sample, and their allocation to the UK Kennel Club breed classifications (gundog, hound, pastoral, toy, terrier, utility and working; Supplementary Tables 1, 2).

**Table 1:** Descriptive statistics of the subjects, including sex, age in months, weight, and height information displayed by breed group (pure breed and mixed/cross breed).

**Breed**	**Total count (%)**	**Sex *N* (%)**		**Age in months (mean ±SD)**	**Weight in kg (mean ±SD)**	**Height in cm (mean ±SD)**
		**Male**	**Female**			
Mixed breeds	417 (34.5)	192 (15.9)	225 (18.6)	97.50 ± 51.05	20.10 ± 11.02	43.41 ± 13.15
Pure breeds	790 (65.5)	365 (30.2)	425 (35.2)	89.8 ± 48.36	21.13 ± 13.88	43.56 ± 15.33
Chi square test/unpaired *t*-test		Chi-squared = 0.003, *p* = 0.958		*t* = 2.58, *df* = 1,205, *p* = 0.010	*t* = 1.31, *df* = 1,205, *p* = 0.190	*t* = 0.18, *df* = 1,205, *p* = 0.854
**Grand total**	1,207	557 (46.1)	650 (53.9)	92.46 ± 49.42	20.77 ± 12.97	43.51 ± 14.61

*A Chi-squared test was run to examine whether the proportion of males and females differed between the two breed groups, and unpaired t-tests were conducted to look for mean group differences in age, weight, and height*.

**Table 2:** Description of categorical questions concerning the dogs and their owners (*N* = 1,207), and percentage breakdown of the groups.

**Title and description**	**Categorical variable labels:**	***N***	**%**
**Age group**	Group one: 1–3 years	185	15.3
	Group two: >3–6 years	251	20.8
	Group three: >6–8 years	191	15.8
	Group four: >8–10 years	202	16.7
	Group five: >10–12 years	170	14.1
	Group six: >12 years	208	17.2
**Neuter status**	Intact	529	43.8
	Neutered	678	56.2
**Sensory problems**	None	980	81.2
	Vision and/or hearing	227	18.8
**Off-leash activity:** how long does your dog walk/run around outdoors without a leash on a typical day?	Less than 30 min	164	13.6
	30 min−1 h	269	22.3
	>1–3 h	367	30.4
	>3–7 h	165	13.7
	More than 7 h	242	20.0
**Body condition score (BCS):** what body shape does your dog have?	Thin (BCS 1–2)	203	16.8
	Normal (BCS 3)	784	65.0
	Over-weight (BCS 4–5)	220	18.2
**Food:** what food are you currently feeding your dog for its main meal?	Dry food only	267	22.1
	Tinned &/or dry food	147	12.2
	Cooked food	306	25.4
	Mixed	294	24.4
	Raw meat	193	16.0
**Vitamins:** do you give your dog vitamins or supplements?	Almost never	328	27.2
	Rarely	391	32.4
	Often	244	20.2
	Regular (daily)	244	20.2
**Trauma:** has the dog experienced a traumatic event, which could still have an effect on it?	No	694	57.5
	Yes	513	42.5
**Health Problems:** what kind of health problems does your dog have?	None	479	39.7
	Tooth problems only	182	15.1
	Joint problems + tooth problems	126	10.4
	Joint problems only	246	20.4
	Other disorders	174	14.4
**Medication:** is your dog currently taking any medication?	No	1,021	84.6
	Yes	186	15.4
Owner age	<29 years	385	31.9
	30–39 years	343	28.4
	40–49 years	253	21.0
	>50 years	226	18.7
**Owner experience:** how would you evaluate your experience with dogs?	Dogs are my hobby/profession and/or I am a dog trainer/breeder	307	25.4
	I have had a dog before	639	52.9
	I had never had a dog before	261	21.6
**Other dogs in household:** how many other dogs do you have living in your household? (Not including this one).	None	433	35.9
	One	474	39.3
	Two or more	300	24.9
**People in household:** how many people are living in the household?	One person (myself)	141	11.7
	Two people	503	41.7
	Three people	271	22.4
	Four or more people	292	24.2
**Child:** do you have a child/children living in your household?	No	919	76.1
	Yes	288	23.9
Age of the dog when arrived: the age of the dog when it arrived in the Owner's household	Less than 7 weeks	265	22.0
	7–12 weeks	530	43.9
	3–12 month	198	16.4
	More than 1 year	214	17.7
**Get dog:** how did you get your dog?	I found it/got it from a shelter	340	28.2
	It was born at my place/bought it	544	45.1
	I got it as a present	323	26.8
**Where dog is kept:** where do you keep your dog?	Outside/inside house with garden	149	12.3
	In a fenced garden	384	31.8
	Urban/Suburban apartment	674	55.8
**Dog obedience tasks:** which tasks can your dog reliably perform? (e.g., sit, lie down, come, fetch, stay, walk at heel, leave/drop it, watch me etc.). Open question.	Maximum a task	151	12.5
	2 kinds of tasks	169	14.0
	3 kinds of tasks	228	18.9
	4 or more kinds of tasks	659	54.6
**Play:** on an average day, how much time do you or other people spend together with your dog in different activities? (Play, walking, training)	Less than 30 min	122	10.1
	30 min−1 h	378	31.3
	>1–3 h	551	45.7
	More than 3 h	156	12.9
**Commands:** how many commands can your dog execute reliably?	<10 commands	540	44.7
	11–30 commands	535	44.3
	>30 commands	132	10.9
**Dog training activities:** how many activities are you currently doing with your dog?	One activity	385	31.9
	2–3 activities	527	43.7
	4 or more activities	295	24.4
**Time spent alone:** how much time does your dog spend alone on an average working day?	None	169	14.0
	1–2 h	276	22.9
	3–8 h	594	49.2
	More than 8 h	168	13.9
**Dog behavior changed:** has your dog's behavior changed over the last 3 months?	No	910	75.4
	Yes	297	24.6

### Procedure

The “Demographic Questionnaire” collected basic information regarding the demographic attributes of the dog, the owner and social attributes of their interactions. Three continuous variables were collected: the current weight (in kg) of the dog, height at the shoulder (in cm), and age (in months; Table 1). The rest of the variables were categorical, and the main descriptive statistics of the subset of 1,207 dogs and their owners are presented in Table 2. Owners were provided with a diagram to help them to classifying their dog's body condition score (please see questionnaire in Supplementary Materials). Dogs were divided into two breed groups; Mixed (including cross breeds) and Pure breeds. In addition to reporting the age in months of the dogs, we also allocated the dogs to six age groups, which would allow us to examine non-linear relationships with age (Table 2). Each separate category of each variable contains at least 10% of the sample. In cases where fewer dogs were allocated, categories were collapsed. Unfortunately, owner gender was not possible to analyze, due to the fact that only 109 male owners filled in the questionnaire, which made up only 9% of the sample. In addition, we were not able to examine individual breeds of dog, as none of the breeds in the sample exceeded 10% of the overall sample, or indeed, 10% of the pure breed sample. The most popular breeds in descending order included the Labrador retriever (*N* = 59, 7.5% pure breed sample, 4.9% overall sample), Hungarian Vizsla (*N* = 58, 7.3%, 4.8), Golden retriever (*N* = 41, 5.2%, 3.4%), Yorkshire terrier (*N* = 36, 4.6%, 3.0), Dachshund (*N* = 35, 4.4%, 2.9%), German shepherd (*N* = 35, 4.4%, 2.9%), Bichon Havanese (*N* = 34, 4.3%, 2.8%), Border collie (*N* = 34, 4.3%, 2.8%), Beagle (*N* = 25, 3.2%, 2.1%), and West highland terrier (*N* = 24, 3.0%, 2.0%). According to the HGV (Heti Világgazdaság) a Hungarian weekly economic and political magazine, in 2017 the top 10 dog breeds in Hungary included the German shepherd, French bulldog, English bulldog, Yorkshire terrier, Vizsla, Dachshund, American Staffordshire terrier, Chihuahua, Boxer, and Golden retriever (54).

### Statistical analysis

#### Descriptive statistics

In order to determine whether the two breed groups (pure breed and mixed/cross bred) differed in sex, age, weight, and height a Chi squared test and Unpaired *t*-tests were conducted. Additionally we highlighted some of the main descriptive statistics of the demographic variables of the sample in the results.

#### Differences in owner and dog demographics in the six dog age groups

Utilizing the reduced dataset of 1,207 individuals, to determine whether certain owner and dog demographics differ according to the age category of the dog, we ran univariate analyses [Kruskal-Wallis tests (continuous variables) and Chi-squared tests (categorical variables)] on the demographic variables by dog age group. In order to take into account multiple comparisons, we used the Benjamini–Hochberg procedure, which controls the false discovery rate (FDR, the expected proportion of false discoveries among all discoveries) and adjusts the *p*-values accordingly (55).

#### Differences in owner and dog demographics in healthy and unhealthy dogs

In order to examine the health status of the dogs, a new variable was produced by combining sensory problems and health problems data. Our intention was to create a variable that reflected health status. Healthy dogs were defined as free from sensory problems, and health problems such as allergies, teeth and joint problems, dysplasia, epilepsy, reproductive issues, heart failure, diabetes, thyroid problems, cancer and infections (56). All dogs, which did not suffer from health or sensory problems were given the value “1,” and the rest received “0.” The new variable was labeled “Health status,” and 39.4% of the sample were “healthy dogs,” leaving 60.6% categorized as “unhealthy.” This binary variable was used as the response variable in a Generalized linear model (with logit link function) that was performed in SPSS v. 22, to identify the key variables associated with health status. Weight and height were included as covariates, and the demographic variables as fixed factors [age group, breed, sex, neuter status, off-leash activity, body condition score, food, vitamins, trauma, medication, owner age, owner experience, how many other dogs in household, how many people in household, child, dog age when arrived, get dog, where dog is kept, dog obedience tasks, play, commands, dog training activities, time spent alone, and dog behavior changed (for descriptions of categories see Table 2)]. Due to the large number of predictors used in the model (26 demographic factors), we only tested for the 2-way interactions with age group: of breed (because we expected that mixed breeds would be even more healthier with age than pure breeds), weight, and body condition score (three factors that have been found to influence health), and the interaction between sex and neuter status, otherwise only the main effects were analyzed. We used a robust model based estimator, as it provides a consistent estimate of the covariance. Non-significant interactions were removed from the model (*p*-values below 0.05), but all main effects that had previously been determined to vary by age group, were left in the model, even non-significant ones. Time spent alone, child, owner experience and how many dogs/people in household were not significant, and since they did not vary with age group, they were removed from the final model. Due to the large number of factors retained in the model, the Benjamini–Hochberg procedure was again utilized to control for the false discovery rate [FDR, (55)]. Most of the categorical variables used were ordinal, which allowed group comparisons to the highest level within that category. The reference category used for age group was the oldest category (dogs aged >12 years), and for body condition score a normal body condition score (3) was used. Parameter estimate results for the full model are presented in the Supplementary Material.

## Results

### Descriptive statistics

The descriptive statistics are presented in Table 2. Here we highlight some of the findings. The two breed groups (mixed and pure breeds) did not differ in sex ratio, weight in kilograms, or height in cm, however, their distribution patterns differed to some extent; pure breeds showed a wider range of body weight and height in cm than mixed breeds. Perhaps due to the fact that pure breed dogs are bred specifically to show more extreme characteristics. Additionally, the mixed breeds sample were on average older than the pure breed sample (Table 1). Nineteen percent of the sample (*N* = 227) suffered from sensory problems (hearing and/or sight issues), 15% were currently taking medication (*N* = 186), and 46% suffered from tooth and/or joint problems (*N* = 554). Fifty percent of dogs were fed cooked or mixed combinations of food (table scraps, cooked/tinned/dry and raw meat, *N* = 600), and 40% received vitamins often/daily (*N* = 488). The owners scored their dogs as having a “normal” body condition in the majority of the sample (65%, *N* = 784), and 34% of the dogs received more than 3 h of off-leash activity a day (*N* = 407). Surprisingly, owners reported that 43% of the dogs had previously experienced a traumatic event (*N* = 513).

Sixty percent of the dog owners were aged under 39 years (*N* = 728), 78% had previous dog ownership experience (*N* = 946), 42% lived with one other person (*N* = 503), and 36% had single dog households (*N* = 433). Fifty six percent of dogs lived in urban/suburban apartments (*N* = 674); however, 32% of dogs were kept in a fenced garden (*N* = 384), which reflects the common country practice of keeping dogs outside, and 63% of dogs were left alone for more than 3 h a day (*N* = 762). Sixty six percent of owners obtained their dog aged under 12 weeks (*N* = 795), 45% got them from a breeder, or the dog was born at their own home (*N* = 544), and 25% of dogs had changed their behavior over the last 3 months (*N* = 297).

Finally, four variables measured owner dog interactions including dog obedience tasks, play, commands, and dog training activities. Fifty five percent of dogs could currently reliably perform four or more tasks (such as sit, lie down, come, and fetch, *N* = 659), 45% knew fewer than 10 commands (*N* = 540), 59% participated in more than 1 h of activity (play, walking, and training) per day (*N* = 707), and 76% participated in two or more dog training activities (*N* = 822).

#### Differences in owner and dog demographics in the six dog age groups

Univariate analysis revealed that 20 of the 27 demographic variables differed significantly between the dog age groups after correcting for multiple comparisons (please refer to Table 3 for details). The oldest age group of dogs (above 12 years) were characterized by fewer pure breeds and fewer females than would be expected by chance. Additionally, this age group had a higher number of dogs with sensory problems, that received daily vitamins, had joint problems and/or tooth problems, were on medication, whose behavior had changed in the past 3 months, and fewer dogs with a normal body condition score. This age group also had a higher number of dogs that lived outside or inside a house with a garden, and received less than 30 min of off-leash activity per day. All four of the dog/owner interaction training variables (dog obedience tasks, play, commands and dog-training activities) were strongly influenced by age in the oldest age group in particular. This age group had more dogs than expected that participated in maximum one dog obedience task, received less than 30 min of play/activity with owner, knew fewer than 10 commands, and participated in only one or no dog training activities.

**Table 3:** The proportion of the dogs present in each category of the categorical variables (with the percentage expected by chance in brackets), presented separately for each dog age group.

**Factor**	**Category**	**Age group (years)**						**Statistics**
		**1–3 *N* = 185**	**>3–6 *N* = 251**	**>6–8 *N* = 191**	**>8–10 *N* = 202**	**>10–12 *N* = 170**	**>12 *N* = 208**	
Height in cm (median ± SD)		45 (15)	43 (15)	45 (14)	43 (15)	43 (14)	41 (14)	Kruskal-Wallis = 11.370 *P* = 0.055
Weight in kg (median ± SD)		20 (13)	17 (13)	22 (14)	20 (13)	20 (12)	18 (11)	Kruskal-Wallis = 4.666 *P* = 0.515
Breed	Mixed	**28 (35)**_a_	37 (35)_a_	28 (35)_a_	35 (35)_a_	35 (35)_a_	**42 (35)**_a_	χ(5) = 12.997
	Pure	**72 (65)**_a_	63 (65)_a_	72 (65)_a_	65 (65)_a_	65 (65)_a_	**58 (65)**_a_	*P =* 0.031
Sex	Female	58 (54)_ab_	50 (54)_ab_	59 (54)_b_	56 (54)_ab_	58 (54)_ab_	**44 (54)**_a_	χ(5) = 14.612
	Male	42 (46)_ab_	50 (46)_ab_	41 (46)_b_	44 (46)_ab_	42 (46)_ab_	**56 (46)**_a_	*P =* 0.019
Neuter status	Intact	**56 (44)**_a_	37 (44)_b_	46 (44)_ab_	44 (44)_ab_	39 (44)_b_	43 (44)_ab_	χ(5) = 18.550
	Neutered	**44 (56)**_a_	63 (56)_b_	54 (56)_ab_	56 (56)_ab_	61 (56)_b_	57 (56)_ab_	*P =* 0.004
Sensory problems	None	98 (81)_a_	94 (81)_ab_	96 (81)_a_	86 (81)_bc_	78 (81)_c_	**36 (81)**_d_	χ(5) = 375.994
	Hearing/vision	2 (19)_a_	6 (19)_ab_	4 (19)_a_	14 (19)_bc_	22 (19)_c_	**64 (19)**_d_	*P* < 0.001
Off-leash activity	Less than 30 min	11 (14)_ab_	14 (14)_ab_	8 (14)_b_	12 (14)_ab_	15 (14)_ab_	**21 (14)**_a_	
	30 min−1 h	25 (22)_a_	21 (22)_a_	20 (22)_a_	25 (22)_a_	22 (22)_a_	22 (22)_a_	
	>1–3 h	**22 (30)**_a_	32 (30)_ab_	32 (30)_ab_	36 (30)_ab_	32 (30)_ab_	27 (30)_ab_	χ(20) = 38.333
	>3–7 h	18 (14)_a_	14 (14)_a_	13 (14)_a_	12 (14)_a_	12 (14)_a_	13 (14)_a_	
	More than 7 h	25 (20)_ab_	19 (20)_ab_	**27 (20)**_b_	14 (20)_ab_	19 (20)_ab_	17 (20)_ab_	*P =* 0.014
Body condition score	1–2 (thin)	**24 (17)**_a_	17 (17)_ab_	15 (17)_ab_	13 (17)_ab_	11 (17)_b_	21 (17)_ab_	χ(10) = 42.492
	3 (normal)	69 (65)_a_	68 (65)_a_	63 (65)_a_	67 (65)_a_	64 (65)_a_	**58 (65)**_a_	
	4–5 (overweight)	**5 (18)**_a_	15 (18)_b_	23 (18)_b_	20 (18)_b_	25 (18)_b_	21 (18)_b_	*P* < 0.001
Food	Dry food only	19 (22)_a_	20 (22)_a_	19 (22)_a_	26 (22)_a_	**31 (22)**_a_	19 (22)_a_	
	Tinned & dry food	12 (12)_a_	12 (12)_a_	**8 (12)**_a_	14 (12)_a_	13 (12)_a_	13 (12)_a_	
	Cooked food	**21 (25)**_a_	24 (25)_a_	27 (25)_a_	28 (25)_a_	23 (25)_a_	**29 (25)**_a_	
	Mixed	25 (24)_a_	24 (24)_a_	**28 (24)**_a_	**20 (24)**_a_	25 (24)_a_	25 (24)_a_	χ(20) = 38.530
	Raw meat	**23 (16)**_a_	21 (16)_ab_	17 (16)_abc_	11 (16)_bc_	**9 (16)**_c_	13 (16)_abc_	*P =* 0.014
Vitamins	Almost never	26 (27)_ab_	30 (27)_b_	27 (27)_ab_	32 (27)_b_	29 (27)_ab_	**18 (27)**_a_	
	Rarely	34 (32)_a_	37 (32)_a_	31 (32)_a_	30 (32)_a_	**26 (32)**_a_	35 (32)_a_	χ(15) = 29.529
	Often	20 (20)_a_	20 (20)_a_	22 (20)_a_	16 (20)_a_	**25 (20)**_a_	19 (20)_a_	
	Regularly (daily)	20 (20)_ab_	13 (20)_b_	20 (20)_ab_	22 (20)_ab_	20 (20)_ab_	**28 (20)**_a_	*P =* 0.021
Trauma	No	**72 (57)**_a_	58 (57)_b_	51 (57)_b_	50 (57)_b_	61 (57)_ab_	54 (57)_b_	χ(5) = 24.260
	Yes	**28 (43)**_a_	42 (43)_b_	49 (43)_b_	50 (43)_b_	39 (43)_ab_	46 (43)_b_	*P* < 0.001
Health Problems	None	**74 (40)**_a_	56 (40)_b_	48 (40)_b_	27 (40)_c_	21 (40)_cd_	11 (40)_d_	
	Tooth problems only	**2 (15)**_a_	9 (15)_ab_	13 (15)_bc_	22 (15)_cd_	26 (15)_d_	20 (15)_cd_	
	Joint & tooth problems	1 (10)_a_	3 (10)_ab_	5 (10)_ab_	8 (10)_bc_	17 (10)_cd_	**29 (10)**_d_	
	Joint problems only	16 (20)_a_	17 (20)_a_	17 (20)_a_	24 (20)_a_	22 (20)_a_	**27 (20)**_a_	χ(20) = 372.200
	other disorders	**8 (14)**_a_	16 (14)_ab_	17 (14)_ab_	19 (14)_b_	14 (14)_ab_	13 (14)_ab_	*P* < 0.001
Medication	No	95 (85)_a_	90 (85)_ab_	87 (85)_ab_	84 (85)_b_	84 (85)_b_	**69 (85)**_c_	χ(5) = 60.620
	Yes	5 (15)_a_	10 (15)_ab_	13 (15)_ab_	16 (15)_b_	16 (15)_b_	**31 (15)**_c_	*P* < 0.001
Owner age	≤ 29 years	**49 (32)**_a_	34 (32)_b_	31 (32)_b_	25 (32)_b_	26 (32)_b_	26 (32)_b_	
	30–39 years	**23 (28)**_a_	30 (28)_a_	28 (28)_a_	33 (28)_a_	25 (28)_a_	30 (28)_a_	χ(15) = 55.070
	40–49 years	**15 (21)**_a_	24 (21)_a_	24 (21)_a_	21 (21)_a_	22 (21)_a_	20 (21)_a_	
	>50 years	13 (19)_ab_	12 (19)_b_	17 (19)_abc_	21 (19)_abc_	**27 (19)**_c_	25 (19)_ac_	*P* < 0.001
Owner experience	Hobby/profession	24 (25)_a_	22 (25)_a_	22 (25)_a_	**30 (25)**_a_	26 (25)_a_	29 (25)_a_	
	Had a dog before	51 (53)_a_	55 (53)_a_	56 (53)_a_	49 (53)_a_	54 (53)_a_	52 (53)_a_	χ(10) = 7.992
	Never had a dog	25 (22)_a_	23 (22)_a_	22 (22)_a_	21 (22)_a_	20 (22)_a_	19 (22)_a_	*P* = 0.643
How many other dogs in household	0	**42 (36)**_a_	40 (36)_a_	37 (36)_a_	30 (36)_a_	35 (36)_a_	32 (36)_a_	χ(10) = 16.675
	1	38 (39)_a_	40 (39)_a_	42 (39)_a_	41 (39)_a_	39 (39)_a_	37 (39)_a_	
	2 or more	21 (25)_a_	20 (25)_a_	21 (25)_a_	29 (25)_a_	26 (25)_a_	**32 (25)**_a_	*P* = 0.101
How many people in household	1	10 (12)_a_	9 (12)_a_	12 (12)_a_	13 (12)_a_	15 (12)_a_	11 (12)_a_	
	2	43 (42)_a_	47 (42)_a_	42 (42)_a_	35 (42)_a_	38 (42)_a_	43 (42)_a_	χ(15) = 13.469
	3	21 (22)_a_	21 (22)_a_	24 (22)_a_	27 (22)_a_	20 (22)_a_	22 (22)_a_	
	4 or more people	26 (24)_a_	23 (24)_a_	21 (24)_a_	25 (24)_a_	26 (24)_a_	24 (24)_a_	*P* = 0.609
Child	No	75 (76)_a_	80 (76)_a_	76 (76)_a_	73 (76)_a_	75 (76)_a_	76 (76)_a_	χ(5) = 3.373
	Yes	25 (24)_a_	20 (24)_a_	24 (24)_a_	27 (24)_a_	25 (24)_a_	24 (24)_a_	*P* = 0.643
Dog age when arrived	Less than 7 weeks	21 (22)_a_	21 (22)_a_	21 (22)_a_	23 (22)_a_	24 (22)_a_	23 (22)_a_	
	7–12 weeks	**56 (44)**_a_	44 (44)_ab_	48 (44)_ab_	39 (44)_b_	39 (44)_b_	38 (44)_b_	χ(15) = 37.476
	3–12 month	18 (16)_a_	18 (16)_a_	14 (16)_a_	15 (16)_a_	16 (16)_a_	17 (16)_a_	
	More than 1 year	**5 (18)**_a_	16 (18)_b_	17 (18)_b_	23 (18)_b_	21 (18)_b_	23 (18)_ab_	*P =* 0.002
Get dog	I found it/from shelter	22 (28)_a_	31 (28)_a_	21 (28)_a_	33 (28)_a_	29 (28)_a_	32 (28)_a_	
	It was born at my place/I bought it	**55 (45)**_a_	46 (45)_ab_	50 (45)_ab_	40 (45)_b_	39 (45)_b_	40 (45)_ab_	χ(10) = 23.066
	I got it as a present	23 (27)_a_	23 (27)_a_	29 (27)_a_	28 (27)_a_	32 (27)_a_	27 (27)_a_	*P =* 0.019
Where dog is kept	Outside/inside house with garden	8 (12)_a_	10 (12)_ab_	12 (12)_ab_	15 (12)_ab_	10 (12)_ab_	**19 (12)**_b_	
	In a fenced garden	35 (32)_a_	**25 (32)**_a_	36 (32)_a_	29 (32)_a_	34 (32)_a_	34 (32)_a_	χ(10) = 24.942
	Suburban/urban apartment	58 (56)_ab_	64 (56)_b_	52 (56)_ab_	56 (56)_ab_	56 (56)_ab_	**47 (56)**_a_	*P =* 0.030
Dog obedience tasks	Maximum a task	10 (13)_a_	7 (13)_a_	12 (13)_a_	10 (13)_a_	12 (13)_a_	**25 (13)**_b_	
	2 kind of tasks	13 (14)_ab_	13 (14)_ab_	**8 (14)**_b_	14 (14)_ab_	17 (14)_ab_	19 (14)_a_	χ(15) = 59.777
	3 kind of tasks	17 (19)_a_	23 (19)_a_	16 (19)_a_	20 (19)_a_	16 (19)_a_	19 (19)_a_	
	4 or more kinds of tasks	60 (55)_a_	57 (55)_a_	63 (55)_a_	56 (55)_a_	54 (55)_a_	**38 (55)**_b_	*P* < 0.001
Play	Less than 30 min	2 (10)_a_	5 (10)_ab_	9 (10)_bc_	12 (10)_bcd_	15 (10)_cd_	**19 (10)**_d_	
	30 min−1 h	30 (31)_a_	28 (31)_a_	28 (31)_a_	34 (31)_a_	34 (31)_a_	**35 (31)**_a_	χ(15) = 61.282
	>1–3 h	52 (46)_ab_	53 (46)_b_	48 (46)_abc_	45 (46)_abc_	39 (46)_ac_	**34 (46)**_c_	
	More than 3 h	16 (13)_a_	13 (13)_a_	15 (13)_a_	**9 (13)**_a_	13 (13)_a_	12 (13)_a_	*P* < 0.001
Commands	<10 commands	47 (45)_ab_	37 (45)_b_	36 (45)_b_	41 (45)_b_	49 (45)_ab_	**60 (45)**_a_	χ(10) = 38.757
	11–30 Commands	44 (44)_abc_	53 (44)_c_	52 (44)_bc_	46 (44)_bc_	38 (44)_ab_	**31 (44)**_a_	
	>30 Commands	9 (11)_a_	10 (11)_a_	12 (11)_a_	13 (11)_a_	13 (11)_a_	**9 (11)**_a_	*P* < 0.001
Dog training activities	One activity	63 (64)_a_	61 (64)_a_	66 (64)_ab_	60 (64)_bc_	61 (64)_c_	**75 (64)**_d_	χ(10) = 162.384
	2–3 activities	25 (19)_ab_	20 (19)_b_	15 (19)_b_	22 (19)_ab_	19 (19)_ab_	**11 (19)**_a_	
	4 or more activities	12 (17)_a_	20 (17)_ab_	19 (17)_bc_	18 (17)_c_	20 (17)_cd_	**14 (17)**_d_	*P* < 0.001
Time spent alone	None	16 (14)_a_	14 (14)_a_	14 (14)_a_	15 (14)_a_	14 (14)_a_	12 (14)_a_	
	1–2 h	22 (23)_a_	19 (23)_a_	25 (23)_a_	26 (23)_a_	23 (23)_a_	23 (23)_a_	χ(15) = 20.849
	3–8 h	52 (49)_a_	53 (49)_a_	48 (49)_a_	47 (49)_a_	41 (49)_a_	52 (49)_a_	
	More than 8 h	10 (14)_a_	14 (14)_ab_	14 (14)_ab_	12 (14)_ab_	**22 (14)**_b_	13 (14)_ab_	*P* = 0.167
Dog behavior changed	No	79 (75)_abc_	88 (75)_c_	83 (75)_bc_	74 (75)_ab_	69 (75)_ad_	**56 (75)**_d_	χ(5) = 72.977
	Yes	21 (25)_abc_	12 (25)_c_	17 (25)_bc_	26 (25)_ab_	31 (25)_ad_	**44 (25)**_d_	*P* < 0.001

*The height and weight variables alone display mean and standard deviation. Where significant group differences were found (indicated by Kruskal-Wallis/Chi-squared tests), the category with the larger or smaller proportion than was expected by chance is marked in bold. P-values were corrected with the Benjamini–Hochberg FDR procedure (significant corrected P-values are marked in italics). Z-tests were performed to compare column proportions (P-values were adjusted for multiple comparison using the Bonferroni method according to the crosstabs procedure in SPSS). Each subscript letter denotes a subset of age categories whose column proportions do not differ significantly from each other at the .05 level*.

Conversely, the youngest age group (dogs aged between 1 and 3 years) had more dogs that were sexually intact, had no health problems or previous trauma, were thin, and were fed raw meat, and fewer that had tooth and joint problems, or other disorders, than would be expected by chance. In addition, this age group contained more owners aged under 29 years, more dogs that were born at the owner's home or bought from a breeder, arrived at 7–12 weeks of age, and fewer dogs that arrived in the household aged more than 1 year, and that knew more than 30 commands.

#### Differences in owner and dog demographics in healthy and unhealthy dogs

The average age of “unhealthy” pure breed dogs was significantly lower than the “unhealthy” mixed breed dogs [unpaired *t*-test: *t* = 2.346, *df* = 729, *P* = 0.019 (Pure breed: mean = 107.27, SD = 45.68, *N* = 485; Mixed breed: mean = 115.91, SD = 49.67, *N* = 246)]. Please refer to the Supplementary Materials for age distribution histograms for healthy and unhealthy dogs separated by breed group (Supplementary Figures 1A,B and 2A,B). A generalized linear model was run to examine the effects of the demographic variables and age group on the Health status variable. Since many of the demographic variables were shown to change according to the age group of the dog, in order to control for age differences, these demographic variables were left in the models (the models were not reduced, apart for the non-significant factors of time spent alone, child, how many people/dogs in the household, and owner experience). Results revealed that there was a significant main effect of age group (Wald Chi-squared = 86.289, *p* < 0.001; FDR *p* < 0.001; Table 4). As dogs aged, the odds of being healthy decreased. Dogs from age group one were 22.8 times more likely to be allocated to the healthy group than dogs in age group six. Age groups one to five were all significantly healthier than age group six. According to the estimated marginal means (after adjustment for the other variables in the model and differences in sample size), 49 percent of dogs aged 1–3 years old (total *N* of age group 1 = 185) were classified as healthy, but only 5% of dogs aged over 12 years (total *N* = 208) received this status.

**Table 4:** Results of the binary generalized linear model with logit link function to examine the demographic variables associated with health status.

**Source**	***df***	***Wald Chi-square***	***P-value***	**FDR**
Age group	**5**	**86.289**	**0.000**	**0.000**
Height (in cm)	1	5.530	**0.019**	0.070
Weight (in kg)	1	2.386	0.122	0.210
Breed	1	2.364	0.124	0.210
Sex	1	1.559	0.212	0.292
Neuter status	1	0.633	0.426	0.493
Off-leash activity	4	10.140	**0.038**	0.105
Body condition score	2	0.059	0.971	0.971
Food	4	5.149	0.272	0.352
Vitamins	**3**	**14.392**	**0.002**	**0.011**
Trauma	**1**	**12.078**	**0.001**	**0.007**
Medication	**1**	**36.708**	**0.000**	**0.000**
Owner age	3	8.617	**0.035**	0.105
Age of dog when arrived	3	6.864	0.076	0.152
Get dog	2	3.151	0.207	0.292
Where dog is kept	3	3.614	0.164	0.258
Dog obedience tasks	3	1.546	0.672	0.704
Play	3	3.606	0.307	0.375
Commands	2	0.806	0.668	0.704
Dog training activities	2	5.328	0.070	0.152
Dog behavior changed	1	3.272	0.070	0.152
Age group ^*^Body condition score	**10**	**23.168**	**0.010**	**0.044**

*Time spent alone, child, how many people/dogs in household and owner experience were not significant, and since they did not vary with dog age group, they were removed from the model. P-values were corrected with the Benjamini–Hochberg FDR procedure (significant values are marked in bold)*.

Unsurprisingly, dogs that were classified as unhealthy were more likely to receive medication and vitamin supplements (Table 5). Dogs that had a history of previous trauma were significantly more likely to be allocated to the unhealthy group (odds ratio 1.82; Table 5).

**Table 5:** Results of the binary generalized linear model with logit link function showing the direction and magnitude of effects (odds ratio and confidence interval), and the significance level of the terms in the demographic variables associated with health status.

**Predictors**	***df***	***Wald Chi-square***	***P-value***	**Odds ratio [Exp(B)]**	**Confidence interval**	**Estimated marginal means**
**Age group**	**5**	**86.289**	**0.000**			
One: 1–3 years		50.161	**0.000**	22.84	9.61, 54.27	0.49
Two: >3–6 years		45.910	**0.000**	16.05	7.12, 35.83	0.24
Three: >6–8 years		24.739	**0.000**	7.62	3.42, 16.96	0.27
Four: >8–10 years		15.162	**0.000**	4.97	2.22, 11.15	0.08
Five: >10–12 years		4.051	**0.044**	2.36	1.02, 5.43	0.08
Six: >12 years		–	–	–	–	0.05
**Height (in cm)**	**1**	**5.530**	**0.019**	1.02	1.00, 1.04	
**Vitamins**	**3**	**14.392**	**0.002**			
Almost never		10.76	**0.001**	2.20	1.37, 3.51	0.24
Rarely		1.427	0.232	1.31	0.84, 2.04	0.16
Often		0.295	0.587	1.14	0.70, 1.86	0.14
Regularly (daily)		–	–	–	–	0.12
**Trauma–No**	**1**	**12.078**	**0.001**	1.82	1.30, 2.56	0.20
Yes		–	–	–	–	0.12
**Medication–No**	**1**	**36.708**	**0.000**	7.97	4.07, 15.61	0.35
Yes		–	–	–	–	0.06
Age group ^*^Body condition score	**10**	**23.168**	**0.010**			
Age group 1^*^Underweight		0.001	0.972	0.97	0.21, 4.60	0.52
Age group 1^*^Overweight		0.336	0.562	0.59	0.10, 3.45	0.53
Age group 1^*^Normal		–	–	–	–	0.43
Age group 2^*^Underweight		2.750	0.097	0.29	0.07, 1.25	0.19
Age group 2^*^Overweight		4.900	**0.027**	0.21	0.05, 0.83	0.22
Age group 2^*^Normal		–	–	–	–	0.34
Age group 3^*^Underweight		0.174	0.677	0.72	0.16, 3.46	0.21
Age group 3^*^Overweight		0.013	0.910	1.09	0.27, 4.42	0.41
Age group 3^*^Normal		–	–	–	–	0.20
Age group 4^*^Underweight		1.005	0.316	0.44	0.09, 2.17	0.10
Age group 4^*^Overweight		9.125	**0.003**	0.09	0.02, 0.43	0.04
Age group 4^*^Normal						0.14
Age group 5^*^Underweight		0.015	0.903	1.11	−1.68, 1.93	0.12
Age group 5^*^Overweight		1.982	0.159	0.34	0.74, 1.53	0.06
Age group 5^*^Normal		–	–	–	–	0.07
Age group 6^*^Underweight		–	–	–	–	0.05
Age group 6^*^Overweight		–	–	–	–	0.08
Age group 6^*^Normal		–	–	–	–	0.03

*The reference category used from health status was “unhealthy” and the response was “healthy.” Significant P-values (in bold) indicate which group differs from the reference value in the respective analysis (The reference value for categorical variables was set to the last category in the group, and is denoted by “–”). The estimated marginal means are the unweighted mean response for each category, adjusted for the other variables in the model (the covariates in the model are fixed at the following values: height = 43.51; weight = 20.77), and are used when comparing across different sample sizes by accounting for each mean in proportion to its sample size*.

**Figure 1:**
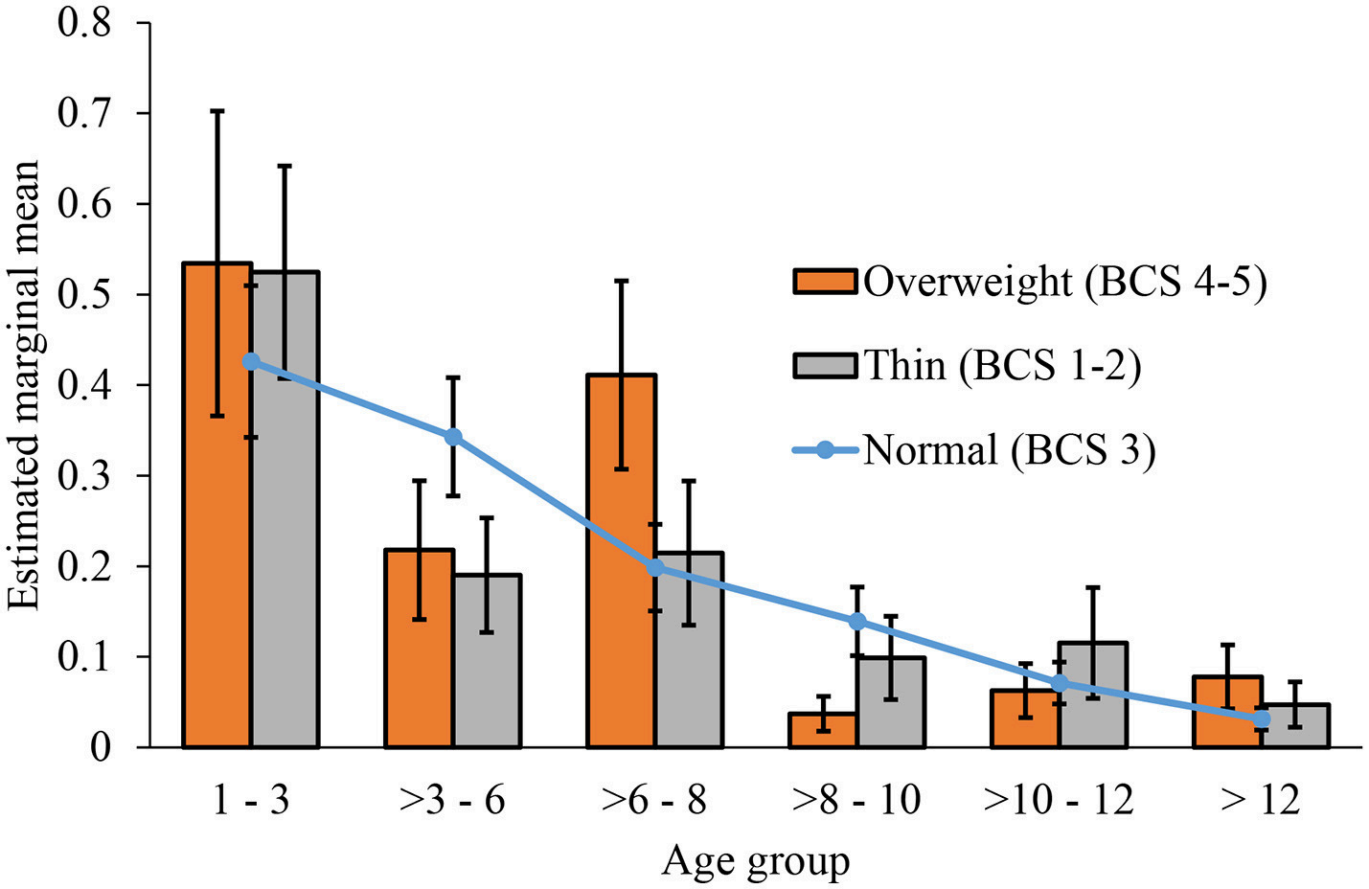
Estimated marginal means of healthy dogs in each age category of the body condition score (BCS). The normal category is used as a reference.

Additionally, there was a significant interaction between age group and body condition score (*P* = 0.010; FDR *p* = 0.044; Table 4; Figure 1). Dogs of normal body weight were compared to dogs that were overweight (body condition score of 4–5) and underweight (body condition score of 1–2). Being overweight was associated with increased odds of being allocated to an “unhealthy” status in the 3–6 years and 8–10 years age groups (Table 5; Figure 1); however, being underweight did not have a significant effect on health status.

Interestingly, when controlling for all other demographic factors, there was a trend for dogs' height in cm to be significantly associated with health status. As height in cm increased, the probability of a healthy status also increased (*P* = 0.019; FDR *p* = 0.070; Table 4). However, the magnitude of the effect was very low (odds ratio = 1.02; Supplementary Materials). Finally, there was a trend for longer daily periods of off-leash activity to be associated with a healthy status, and dogs of owners aged over 50 years were also more likely to be in the “healthy” group, however, these predictors were not significant after FDR correction (Table 4).

#### What factors contributed to dogs trauma status?

We were interested in what type of life events the dog might have lived through, which might have caused trauma to the dog, in order to gain a deeper understanding about the connection between trauma history and current health status. Since we did not specifically ask each owner what event they referred to when they indicated that their dog had experienced a traumatic event, we utilized a second question from the questionnaire “Have any significant changes occurred in the life of your dog?” We examined what specific factors might have contributed to owners' decision to indicate that their dogs had experienced previous trauma, without using the owners subjective opinion as to what specific event might have caused their dogs behavioral changes. The owner could mark multiple possibilities from a list: Mating/giving birth, changes in family structure (for example divorce, birth of children, someone moving away, death in the family), changes in the number of dogs living in the household (new dog arrived, old dog died), moving to a new house, changing owner, changes in the time the dog has to spend alone (e.g., due to a new workplace of owner), the dog has been lost for more than a day, the dog has lived through a traumatic injury/prolonged disease/illness/surgery, and the dog spent time at the shelter. These specific life events were chosen, as they are known to cause stress according to the human and dog literature. The binary variable previous trauma (yes/no) was used as the response variable in a Generalized linear model (with logit link function) that was performed in SPSS v. 22, to identify the key variables associated with trauma status. The binary categories of spent time at a shelter (28% yes, *N* = 340), changed owner (14% yes, *N* = 169), traumatic injury/prolonged disease/surgery (13% yes, *N* = 160), lost for a time (4% yes, *N* = 53), change in family structure (35% yes, *N* = 424), neutered (56% yes, *N* = 678), spent more or less time alone (27% yes, *N* = 327), moved house (32% yes, *N* = 385), number of dogs changed (43% yes, *N* = 523), and pregnancy/mating (14% yes, *N* = 172) were entered into the model as fixed factors. Non-significant factors were removed from the model (*p*-values below 0.05).

Results revealed that there was a significant main effect of spent time at a shelter (Wald Chi-squared = 85.844, *p* < 0.001; Table 6). Dogs that had previously spent time at a shelter were 3.9 times more likely to be allocated to the “trauma” group by owners, than dogs that had not previously spent time at a dog shelter. According to the estimated marginal means (after adjustment for the other variables in the model and differences in sample size), 86 percent of dogs that had spent time at a shelter (*N* = 340) had experienced a traumatic event (according to owner), which had a lasting effect on their behavior (*N* = 235), but 61% of dogs that had never been to a shelter (*N* = 867) also received this status. Dogs' that had previously changed owner, or who had suffered from traumatic injury/prolonged disease/surgery were also more likely to be allocated to the “trauma” group (*P* < 0.001; Table 6). Dogs that were still experiencing negative behavioral consequences of previous traumatic events were significantly more likely to have been lost for a short time, or had experienced a change in the family structure (such as a death or a birth; *P* < 0.03; Table 6). Finally, there was a trend for neuter status to be associated with trauma status, such that dogs that had been neutered were more likely to be in the “trauma” group. The full details from both the full and reduced models are available in the Supplementary Materials.

**Table 6:** Results of the binary generalized linear model with logit link function showing the direction and magnitude of effects (odds ratio and confidence interval), and the significance level of the terms in the life events associated with trauma status.

**Predictors**		***df***	***Wald chi-square***	***P-value***	**Odds ratio**	**Confidence interval**	**Estimated marginal means**
Spent time at a shelter:	Yes	1	85.844	**0.000**	3.87	2.90, 5.15	0.86
	No		–	–	–	–	0.61
Changed owner:	Yes	1	31.625	**0.000**	3.15	2.11, 4.69	0.85
	No		–	–	–	–	0.64
Trauma injury/disease/surgery:	Yes	1	22.464	**0.000**	2.42	1.68, 3.49	0.83
	No		–	–	–	–	0.67
Lost for a time:	Yes	1	5.196	**0.023**	2.19	1.12, 4.31	0.82
	No		–	–	–	–	0.68
Change family structure:	Yes	1	5.858	**0.016**	1.38	1.06, 1.80	0.79
	No		–	–	–	–	0.73

*Significant P-values (in bold) indicate which group differs from the reference value in the respective analysis (The reference value is set to the last category in the group, and is denoted by “–”). Spent more or less time alone, moved house, number of dogs changed, and pregnancy/mating were not significant at P < 0.05 and were removed from the model*.

Since multiple factors were associated with possible trauma in the dogs, we next analyzed whether the culmination of multiple significant factors within individuals predicted trauma status (significant factors included spent time at shelter, changed owner, traumatic injury/prolonged disease/surgery, lost for a time, and change in the family structure). The number of these events per individual dog was summed to create the variable count of significant life events. Next, we made a new categorical variable, “Life events” with four possibilities (“None” the dog had experienced no significantly traumatic events, “One” the dog experienced one event, “Two,” and “Three or more”). We ran a univariate analysis (Chi-squared test) on the “life events” categories per individual by trauma status. Results revealed that three or more life events experienced in the dog's lifetime resulted in a significant increase in the likelihood that dogs experienced negative behavioral consequences, and were allocated to a “trauma” status by the current owner when compared to chance (Table 7).

**Table 7:** The proportion of the dogs present in each category of the categorical variable “life events” (with the percentage expected by chance in brackets), presented separately for dogs in the trauma group (No and Yes).

**Factor**	**Category**	**Previous trauma**		**Statistics**
		**No**	**Yes**	
Life events	None	30 (22)_a_	11 (22)_b_	
	One	38 (32)_a_	25 (32)_b_	
	Two	23 (26)_a_	29 (26)_b_	χ(3) = 162.672
	Three or more	9 (20)_a_	**35 (20)**_b_	*P* < 0.001

*Where a significant group difference was found the category with the larger or smaller proportion than was expected by chance is marked in bold. Z-tests were performed to compare column proportions (P-values were adjusted for multiple comparison using the Bonferroni method). Each subscript letter denotes a subset of previous trauma category whose column proportion did not differ significantly from each other at the 0.05 level*.

## Discussion

In this study, we found numerous differences between the age groups of the dogs in their demographic and dog keeping characteristics: 20 from the 27 comparisons were significant after correcting for multiple comparisons. The oldest age group had fewer pure breeds and females, more dogs with sensory, joint and/or tooth problems, and fewer dogs with a normal body condition score. This group also received daily vitamins and medications, and their behavior had more often changed in the past 3 months. We also found differences in the dog keeping characteristics, e.g., the oldest age group had a higher number of dogs that lived in a house with a garden, and received less than 30 min of off-leash activity per day. Older dogs knew fewer dog obedience tasks and commands, received less than 30 min of play/activity with the owner, and participated in only one or no dog training activities.

Conversely, the youngest age group had more dogs that were sexually intact, were thin, and were fed raw meat, and had no health problems or previous trauma, and fewer that had tooth and joint problems, or other disorders. In addition, this age group contained more owners aged under 29 years, more dogs were born at the owner's home or bought from a breeder, and arrived at 7–12 weeks of age. However, we found no age group difference in the dogs' height, weight, number of dogs/people/presence of children in the household, owner experience or amount of time the dog spent alone.

After controlling for dog and owner demographic variables, dogs that had sensory or health problems were found to be older, required medication and supplements, were more likely to have previously experienced a traumatic event, and be classified as “overweight” in body condition score at certain ages. Our results partially contradict our prediction that dogs that are heavier in weight are more likely to suffer from health problems. However, they also implicate a new factor, which appears to have an impact on health regardless of breed and age, that of experiencing one or more traumatic events at some point during the lifespan. Dogs that had previously spent time at a dog shelter, changed owner, suffered traumatic injury/prolonged disease/surgery, were lost for a time, or who experienced a change in family structure were significantly more likely to be allocated to the trauma group, where the dog's current behavior is thought to be influenced by previous traumatic events. Indeed, two or more of such events experienced in the dog's lifetime resulted in a significant increase in the likelihood of dogs being allocated a “trauma” status. Moreover, since having a “trauma” status was associated with an increased likelihood of health problems/sensory loss, our study contributes to the growing evidence that chronic stress can have negative effects on health and lifespan in the domestic dog. The stress caused by traumatic events results in compromised welfare, and therefore interventions to prevent or alleviate the consequences of trauma should be implemented to improve quality of life in pet dogs.

Many of the demographic differences found seem obvious and are easily explained by basic life history. However, to the best of our knowledge, this is the first time an extensive investigation has been carried out to examine the demographic differences in the life stages of the domestic dog. The presence of fewer pure breeds in the aged dog group (>12 years) corroborates previous research, that found the lifespan of pure breed dogs is lower compared to mixed breed dogs across all weight categories (37, 57, 58). Leading to the suggestion that the artificial breeding of dogs has reduced their life expectancy through increased early mortality and disease risk and early onset of senescence or increased aging rate (59). Sex differences in aging have also been examined, however, some studies have reported a longer mean life span in females (60), and others in males (57, 61). However, neutering has a larger effect on life span than sex (41). Of course, it is also possible that the lower number of females found in the oldest age group in the current study may just be a coincidence, with fewer owners of aged female dogs participating in the online questionnaire than owners of aged male dogs.

The well-known negative correlation between body size and life expectancy was shown only in a tendency for more shorter dogs to be represented in the aged dog population in comparison to young dogs (<12 years mean height at shoulders = 41 cm, and aged 1–3 = 45 cm).

The finding that fewer dogs over 12 years of age had a normal body condition score is consistent with the fact that aged dogs are more likely to suffer from sarcopenia (the decline in skeletal muscle strength and mass), and a decrease in metabolism. Previous studies have found a higher incidence of aged dogs classified as underweight compared to other aged groups (62, 63), which may reflect the presence of undiagnosed or uncontrolled age-related disease, such as age-related sarcopenia. Additionally, as dogs' age, their chances of becoming obese increases, due to a decrease in metabolism, which can result in an increase in body fat mass, if diet type and quantity is not adjusted. The majority of senior dogs experience an overall decrease in energy requirements (64), and an age- or disease-related decline in activity will further reduce energy requirements, additionally increasing the risk of obesity (22).

Age is the greatest risk factor for nearly every major cause of mortality; therefore, it comes as no surprise that aged dogs suffer more from sensory decline, joint and/or tooth problems. Seventy six percent of dogs aged over 12 years suffered from joint and or tooth problems, and 64% had hearing and or vision loss, compared to only 11 percent who did not display age related health problems. The onset of sensory decline appears to be rather sudden, with nearly three times as many dogs reported to suffer from sensory impairments in the oldest age (>12 years) compared to the 10–12 years old group. The prevalence of age related problems explains the higher incidence of vitamin supplementation and medical intervention in these dogs.

Previous studies have shown that not only is the metabolism of aged dogs reduced, but their activity levels and mobility also decline even in the absence of any disease, and are thus a normal part of aging (13). Therefore, the reductions in off-leash exercise, interactive play/activity with the owner, and dog training activities in aged dogs found in the current study, can be explained by a large reduction in the dogs' activity levels, along with a higher occurrence of degenerative joint problems. Loss of muscle strength and function not only decreases mobility and quality of life, but also is related to numerous unfavorable health outcomes. Sarcopenia is a major determinant of impairment, disability, and longevity, and occurs whether or not obesity is present. Sarcopenia is thought to occur due to a decline in resistance-type physical activities. Nutritional interventions combined with physical therapy to increase muscle mass and strength have been found to halt or even reverse sarcopenia in humans (65, 66), and therefore can increase longevity. However, to date we have found no studies that have examined pharmaceutical, nutritional, or exercise related interventions in dogs to prevent/holt the deleterious effects of sarcopenia (67).

The fact that dog obedience tasks and the number of commands known by the dog were at their lowest levels in the oldest age group, indicates either that this age cohort received less training from their owners throughout their lives, or they gradually forgot the commands/tasks due to reduced/absent training in later life, or due to failing memory/advancing dementia. The prevalence of neurodegenerative cerebral changes and associated impairment of cognitive functions, which are not normal and cannot be explained by other medical conditions increases in range from 14 to 35% in dogs more than 8 years of age (68). Canine Cognitive Dysfunction shares multiple similarities to human dementia of the Alzheimer's type, and targeted programs promoting mental exercise and nutritional supplements may be used to delay progression once clinical signs have been presented (69–71). Therefore, aged dogs could benefit greatly from increased training in old age, especially if they have reduced mobility, as these dogs have limited exposure to environmental enrichment (72).

On the other hand, the youngest age group of dogs was also associated with factors that deviated from the norm. Many more dogs remained reproductively intact in 1–3 year olds, which may reflect a shifting in attitude toward the beneficial health effects of delayed neutering, or could be due to the higher incidence of pure breeds in this group, since mixed breeds are more likely to be neutered (42). The elevated number of pure breeds in our young dog sample was likely due to the fact that previous studies have established that mixed breeds dogs are more likely to be brought into the household at an older age than pure breeds (42). Alternatively, a recent shift toward the keeping of pure breeds may have occurred in this Hungarian sample.

Younger dogs were more often classified as thin, with a body condition score of 1–2, and were often fed a raw meat diet. The emergence of raw feeding is a relatively new phenomenon, and the potential health benefits and risk effects of long-term feeding of raw meat diets have not been critically evaluated (73). However, since this group of dogs was more often owned by people aged under 29 years, it is likely that the higher number of dogs fed a raw diet is also due to differing attitudes of the younger generation of owners. For some, the act of feeding is a way to enhance and reinforce the dog- human bond, and the increased advertisement of the anecdotal benefits of raw feeding (and the occasional health scares from the dry food industry) have caused a shift toward the perceived more “natural” diet (73).

The results from the health status analysis generally confirm our findings from the demographic age analysis. That is, older dogs are less healthy and subsequently require medication and dietary supplementation. Our results collaborate previous research that indicates that age is the strongest predictor of health status regardless of breed, height and weight (34), as it is in humans (31). Pure breed dogs that were classified as “unhealthy” were on average younger than “unhealthy” mixed breed dogs. This implies that pure breeds are more likely to suffer from health problems at a younger age. Our results corroborate previous studies that concluded that mixed breeds tend to have a longer healthspan than pure breeds (26, 74–76). However, previous research has also determined that breeds differ in longevity and specific types of morbidities; therefore, it is possible that mixed breeds and pure breeds differ in specific types of health problems and inherited disorders (74).

Despite the fact that neuter status and sex have been found to influence health and longevity in dogs (40, 77, 78), we found no evidence for an effect on health status in our sample. Nor did we find a significant effect of weight of the dog on health status, as was predicted. Although there was a slight tendency for heavier dogs to be classified as unhealthy (*P* = 0.122), which might indicate that owners may not have had the correct weight information for their dogs when they completed the survey, or that a larger sample size is necessary to detect an effect. In addition, there was a significant effect of height on health status, with taller dogs more often classified as healthy, than shorter dogs, regardless of body condition score, weight, breed or age. However, this effect did not remain significant after correction for multiple comparisons (FDR *P* = 0.070). Greer et al. (28) found a negative correlation between height and longevity, however, we could find no reference to the relationship between height and health status, although there are several studies that implicate body weight as the more important predictor of health, as discussed previously. The effect size of the relationship in the current study was low, as was the odds ratio increase in health status with height. Nevertheless, we can speculate that taller dogs might have a longer healthspan compared to their lifespan, even though they have a shorter lifespan than smaller dogs. Taller dogs suffered less from some of the health problems in the survey, such as tooth problems, which afflicted 26% of dogs in the sample (18% of dogs over 60 cm in height compared to 36% of short dogs under 25 cm regardless of age). Kyllar and witter (79) documented a higher frequency and earlier onset of periodontal disease in small breed dogs in comparison to large breeds. Recently, studies have shown a close association of dental disorders with the general health of the animal. Periodontal disease is associated with subsequent diagnosis of cardiovascular diseases, and chronic kidney disease (80, 81). Additionally, dogs that have been selectively bred to be dwarfs, as is increasingly becoming the current fashion (for example Pugs, Bulldogs, Dachshunds and Basset Hounds) are known to suffer from enlarged joints, breathing difficulties as a result of their malformed skulls and abnormal nasal passageways, spinal abnormalities, and eye problems. As an alternative explanation of the results in the current study, some owners may have overestimated their dog's height at the withers in the survey (even though we recommended using a tape measure).

Rather than a main effect of body condition score on health status in dogs, we found an interaction between age and body condition score. This indicates that there were specific ages where the body condition of the dog had a stronger association with health status within the cohort. Being overweight was associated with increased odds of sensory and/or health problems in the 3–6 years and 8–10 years age groups. Previous studies have suggested that the prevalence of obesity and overweight is greatest for dogs aged between 6 and 10 years (82). Our findings could help veterinarians to target specific at risk age groups and to implement weight loss plans for affected dogs. Calorific restriction increases median lifespan and delays the onset of chronic disease in dogs (83). Therefore, dogs' health and welfare can be improved by increased public knowledge of the benefits of feeding a low calorie diet. Additional research utilizing cross-sectional and longitudinal samples are necessary to determine whether there is a link between health and body condition score in the specific age groups found in the current study, and the direction of the relationship (whether health condition cause dogs to become less active and therefore gain weight, or vice versa).

Unsurprisingly, dogs that were classified as unhealthy were more likely to receive medication and vitamin supplementation. Forty percent of the entire sample received regular vitamin supplements. Therefore, we can speculate that some dogs received vitamins as a preventative health measure, and not only due to current health status. There is little information available regarding the influence of vitamin supplementation on subsequent health in dogs. However, in humans and dogs supplementation with a combination of the antioxidants vitamin C and E led to improved cognition and promoted healthy brain aging (84, 85), and in the case of the dogs health was improved especially when antioxidant supplementation was combined with behavioral enrichment. Additionally, antioxidants can decrease inflammation and oxidative stress and increase antioxidant capacity in dogs with osteoarthritis (86); thus, helping to provide proof of the benefits of vitamin supplementation on health in dogs.

Interestingly, after correction for multiple comparisons we did not find a significant association of health status with any of the activity related demographic factors (such as off-leash activity, play, or dog training activities). Exercise is known to promote healthy aging in humans (87), and in several study in dogs, lower exercise frequency and duration was found to be strongly associated with overweight status (10, 48, 88). Therefore, we might expect that dogs that receive a higher amount of exercise per week to be healthier. However, additional longitudinal studies are necessary to determine causality and any direction to the causality of the association between exercise, body condition and health status.

Finally, we found an association of the factor “trauma” with health status in dogs. To our knowledge, very few studies have examined the effects of experiencing a traumatic event or multiple events and how it may lead to reduced health. Cannas et al. (89) analyzed the relationship between stress and tumor development in pet dogs. They found that before the cancer diagnosis, dogs faced changes in their household and routine (such as death of an owner, arrival of new family member, changing owner, moving house, and changes in owners' working shift times). Additionally, they often experienced surgery or a traumatic event, and showed signs of stress and anxiety (such as increased fear and aggression behaviors), compared to an age, neuter status and sex matched control group. In the current study, owners tended to rate the following events as traumatic for their dog: spending time in a shelter, changing owners, traumatic injury/prolonged disease/surgery, getting lost, and changes in family structure, such as birth and death of family members. Multiple traumatic events over the lifespan increased the risk that owners reported that their dogs currently showed behavioral signs that they attributed to previous trauma. Similarly, in humans, adverse childhood experiences (ACEs) have been found to have profound negative effects on health and wellbeing over the lifespan, which increases with the number of ACEs experienced (90). We can speculate that exposure to traumatic experiences causes behavioral changes in dogs such as increased fearfulness and aggression to certain stimuli. Repeated exposure to these stressors can lead to elevated levels of stress hormones, immune system disorder and premature aging (91, 92). Behavioral changes in dogs can often become problematic and are the most common reason behind relinquishment (93).

However, some dogs may experience multiple negative events over their lifetime but do not develop any long lasting negative behavioral consequences. This is because personality traits are thought to affect how an individual reacts to the environment and to stressful events (94). Different personality profiles combined with learning from previous experiences result in differing behavioral strategies adopted by the dogs in order to cope with stressful experiences. For example, Horváth et al. (95) described three coping styles utilized by working German Shepherds to a social threat, that of passive, proactive and ambivalent. Personality types (influenced by genetics and early socialization) and individual coping strategies likely interact to determine an individual's resilience—the ability to avoid deleterious behavioral changes in response to chronic stress (96). In non-human animals, highly affiliative/social individuals tend to be healthier and less reactive to stress, whereas individuals who react with emotional reactivity during stressful events show lower immune function (97).

To the best of our knowledge, only one study has examined the impact of traumatic events on subsequent behavioral responses in dogs. Serpell and Duffy (98) reported that particularly frightening or traumatic events during the guide dog puppy-raising period (up to 6 months of age), were associated with specific behavioral outcomes at 12 months of age. Indicating that there are long-term negative consequences of traumatic experiences. In a retrospective study, Dreschel (49) found that dogs that suffered from a fear or anxiety disorder experienced negative effects on health and lifespan. Specifically, dogs that were afraid of strangers had shortened lifespan and anxiety was associated with skin disorders. Indeed, in a study by King et al. (99) after controlling for dogs' age, sex, neuter status, size, and presence of medical problems, owner reported anxiety and impulsivity were significantly associated with premature graying in young dogs (between 2 and 4 years of age), as was age. Since only 7.8% of the sample (*N* = 31/400) had medical problems, future studies with larger sample sizes should investigate any link between premature graying, anxiety and impulsivity and health problems in dogs.

Since stress affects the physical, mental and social health of the animal, managing an animal's stress after a traumatic event, as well as attempting to prevent the occurrence of the event in the first place, is essential in order to improve healthspan and wellbeing in dogs. Owners should be made aware of the personality type of their dog, and the methods they use to cope with stressful experiences, as well as the most common types of trauma and their risk factors. In this way, they can better support their dog when stress is unavoidable, but also can attempt to reduce their dog's exposure to stress in order to diminish any negative impacts on their healthspan. Since personality and coping style can also change with age within an individual, owners should learn how to read their dogs behavior to better understand their specific needs at all life stages, as well as how to prevent the development of unwanted negative behaviors in their dogs using positive training techniques.

In humans, adverse childhood experiences (ACEs), result in increased mortality and decreased healthspan, through increased susceptibility to the development of diseases such as cardiovascular disease, cancer, chronic respiratory disease and diabetes, and increased risk of subsequent unintentional injury and violence (100). Similarly to humans who suffered ACEs, preclinical studies on the effects of early stress in animals show altered neurological development, including reduced anticipatory reward response and impulse control (101–103). Future cross-sectional and longitudinal studies should attempt to quantify and define the differing types of traumatic events experienced by dogs, and clarify whether experiencing trauma results in long-lasting personality changes, reductions in health, learning and memory, reduced anticipatory reward response and impulse control.

This cross-sectional questionnaire study had several limitations. Firstly, the sample size was relatively small for a large-scale demographic analysis, which limited our ability to detect significant associations after correction for multiple comparisons, and to examine interactions between the demographic variables. This was a hypothesis-generating study and therefore a larger confirmatory study is needed in the future. To better understand demographic changes with age group and their influence on health, future studies should examine individual breeds of different ages. Secondly, all of the health problems examined were treated equally, and the presence of any single health problem resulted in dogs receiving the status of “unhealthy.” Futures studies should determine the effect of demographics on specific health problems. Additionally, some morbidities are known to have more severe effects on physiology and health (such as diabetes). Therefore, studies that apply particular weights to health problems, examine chronic and temporary conditions and also multimorbidities, may uncover more associations with demographic variables. Thirdly, owners may under or over report the occurrence of health problems in their dogs (104), or incorrectly state their dog's height/weight or classify their dogs body condition score (8), which could potentially have confounded our results. Previous studies have indicated that owners' consistently underestimate their dogs' body condition score (105, 106), which could explain why we were only able to detect health associations with body condition score in some age groups. However, owners in general seem to be reliable informants of demographic information (107) and of dog behavioral questionnaires (108). Fourthly, dog keeping practices, owners' attitude and perceptions of their dogs, and their dogs behavior may vary around the world, therefore, further studies are necessary to determine whether cross-cultural differences exist (109). Traditionally, Hungarians viewed the dog as a working animal; however, more recently their opinion of dogs has changed, particularly in large cities and urban environments. The Hungarian owners who were motivated enough to fill in our extensive online questionnaire represent these changing attitudes, as in response to the question, “Why did you get your dog,” over 95% of the owners replied that they got their dog as a social partner or for companionship. In another Hungarian general owner demographic questionnaire we are currently analysing, 75% of owners reported that they regarded their dog as a family member. This figure is comparable to a recent report by RSA Insurance group MORE TH > N of 10,000 owners across the UK that found that 78% (or four out of five) owners see their cat or dog as a member of the family (110). Fifthly, due to time restraints we were not able to include questions regarding the owners' education level, occupation and economic circumstances, all factors that are known to influence health in humans (111). Future studies should focus more on owner demographic factors when examining health status in dogs. Finally, due to the correlational nature of the study design, it was not possible to determine the cause and effect of the various association found. However, our results have shown that age and certain demographic factors are associated with health. Further research into these demographic variables could lead to the advancement of canine health management and keeping practices, which would result in significant welfare gains for pet dogs. Finally, longitudinal studies are necessary to gather information about the direction of the relationships found in the current study, in order to determine any protective and/or risk factors for successful aging to provide interventions to increase healthspan and longevity in pet dogs.

## Conclusion

This is the first extensive cross-sectional investigation to examine demographic differences in the life stages of the domestic dog. We found numerous demographic and environmental differences between dog age groups based on the owners' reports, many of which are new to the literature, such as decreased off-leash activity and dog/owner interaction and training in the oldest age group, and increased raw meat feeding in the youngest. These highlight the importance of taking into account owner and dog demographic changes with age, and their possible impact on behavioral, cognitive, and health measures.

In humans, age is the strongest predictor of healthspan, and our results confirm that this is true also in dogs. Pure breed dogs were found to suffer from health problems from a younger age, and may undergo early mortality in comparison to than mixed breeds. Unsurprisingly, after controlling for dog and owner demographic variables, dogs that had sensory or health problems were found to be older, require medication and supplements, and be classified as “overweight” in body condition score at certain ages. Our study is one of the first to report the long-term negative consequences of traumatic experiences on health in dogs. Dogs that had previously experienced a traumatic event that still currently affected their behavior were more likely to suffer from health problems. The high prevalence of dogs that were affected by one or more traumatic life events in their lifetime indicate that welfare and health could be improved by informing owners of the greatest risk factors of trauma and promoting responsible dog ownership. Additionally, there is a need for greater transparency and clear guidelines for owners of dogs of different life stages. For example, many owners are not aware of the existence of nutritional, medical, and nutraceutical treatments, as well as the benefits of cognitive enrichment, weight control, physiotherapy and exercise to improve their dogs' physical and cognitive state, regardless of age or disease status.

## Ethics statement

We collected the data using an online questionnaire designed to assess the dogs and the owners demographic data via owner report. According to the currently operating Hungarian law (1998. Evi XXVIII. Torveny—the Animal Protection Act, 3rd paragraph, 9th point), non-invasive observational data collection on dog demographics and behavior are not considered as animal experiments, and are therefore allowed to be conducted without any special permission from the University Institutional Animal Care and Use Committee (UIACUC). The filling out of the questionnaires was voluntary and anonymous so the study did not violate respondents' privacy. Informed consent was included in the introductory letter of the questionnaires.

## Author contributions

EK and DS conceived and designed the questionnaire. LW, EK, and BE-B analyzed the data. LW, DS, EK, and BE-B interpreted the results. LW wrote the first draft of the paper. LW, EK, and DS revised the paper. EK and DS provided funding.

### Conflict of interest statement

The authors declare that the research was conducted in the absence of any commercial or financial relationships that could be construed as a potential conflict of interest.
