# Supplementary material for: Demographic Change Across the Lifespan of Pet Dogs and Their Impact on Health Status
Source: Front Vet Sci. 2018 Aug 23;5:200. doi: 10.3389/fvets.2018.00200 (PMC6115627; doi:10.3389/fvets.2018.00200)

**Supplementary copy of the Questionnaire**

**Please note that only the questions used in the analysis are presented here. Some additional questions were asked, and these have been used in separate publications.**

**The link to a copy of the Questionnaire in Hungarian can be found here –**

[**https://goo.gl/forms/IIXQPIEQXsa5qcHu1**](https://goo.gl/forms/IIXQPIEQXsa5qcHu1)

**The question’s title, which corresponds to the title in Table 2, is included in each relevant question.**

**Ageing in the dog**

Are you interested in how dogs change, as they grow old? Do you have a dog? Would you like to participate and help science? Please fill out our questionnaire.

The purpose of our research is to explore the nature and background of aging problems, physical and behavioural changes. The studies are being conducted at the ELTE Ethology Department, within the framework of the Senior Family Dog Program supported by the European Research Council.

Although we are primarily interested in old dogs, you can take part in the questionnaire with any age of dog. So even if you have a young dog, please read on.

The questionnaire consists of three parts and it will take 15-20 minutes to complete. It contains questions about your dog's essential characteristics, behaviour, health, living conditions, lifestyle, qualifications and personality.

You should know your dog's weight in kilograms and height in centimetres, as well as details such as their date of birth and the name of the kennel (if the dog is a pedigree). So it is worth looking for your dog’s pet passport / veterinarian logbook, a measuring tape, and if you do not know the weight of your dog, you can use human scales to take the weight of the dog whilst you are carrying him/her and then take your weight off the value to get the weight of the dog.

If you have multiple dogs, please fill out a separate questionnaire for each dog.

We store your data exclusively for the purpose of research and if necessary to contact you and we do not pass it on to third parties.

You can read our privacy policy here: https://kutyaetologia.elte.hu/beleegyezõ- nyilatkozat/

Thank you for your contribution!

kutyaetologia.elte.hu/szenior-csaladi-kutya-program

facebook: facebook.com/groups/szeniorcsaladikutyaprogram/

e-mail: seniordog@ttk.elte.hu, Enikő Kubinyi

1. Your dog’s name*
2. Age group: Date of birth *(yyyy.mm.dd. If you do not know exactly, please estimate the Year and Month as accurately as possible).
3. Name of the owner (unique identifier). * You can use your first name, or a nickname. We use this as an additional way to identify your dog. Please make a note of the identifier you entered here and if you fill in the questionnaire again, use the same identification.
4. Email address (required only for verification, we do not pass it on to third parties).
5. Breed: Dog breed * (Please select from the breeds in the list, but if your dog’s breed is not present, please tick “Other” and specify the breed in the text box).
6. Off-leash activity: How much do you allow your dog to walk/run without a leash outdoors on an average day? * (Please mark only one option)
   1. no more than a few minutes
   2. less than half an hour
   3. 30 mins - 1 hour
   4. 1-3 hours
   5. 3-7 hours
   6. more than 7 hours
7. Height: How many cm is your dog at the height at the top of the shoulders?*
8. Weight: Weight of your dog (in kg)*
9. Body condition score: What kind of body shape does your dog have? * (Please rate your dog’s body shape from 1 to 5. 1 corresponds to being thin, 5 to being very over-weight. Please use the provided picture guideline to determine the body shape of your dog! If your dog has long hair, then please feel under the hair to help determine body shape).


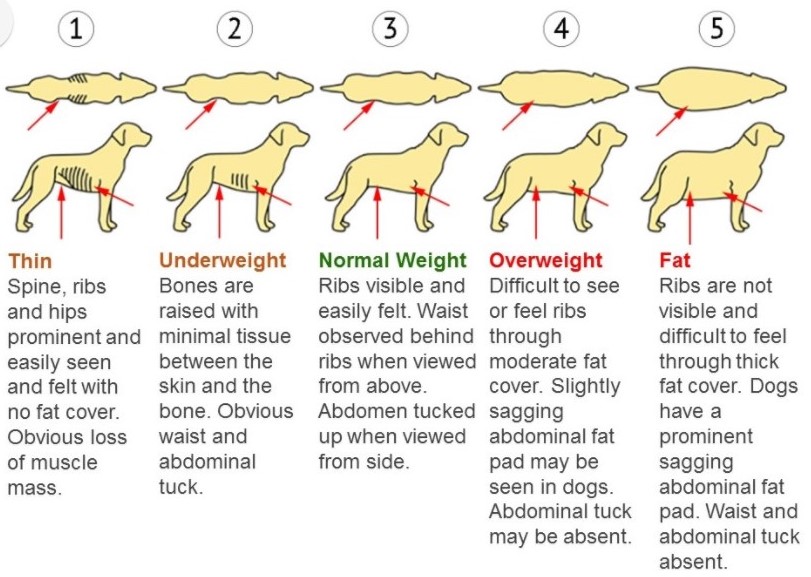


1. Food: What do you feed your dog for his/her main meal? * (Multiple answers can be selected).
   1. Tinned meat
   2. Dry food
   3. Kitchen scraps
   4. Raw meat (BARF)
   5. Home cooked food
   6. Other
2. Play: How much do you and others actively engaged with your dog on an average day (i.e. Playing, walking, and training)? * (Please mark only one answer)
   1. no more than a few minutes
   2. less than half an hour
   3. 30 mins - 1 hour
   4. 1-3 hours
   5. 3-7 hours
   6. more than 7 hours
3. Commands: How many commands does your dog know (and follow)?* (Please mark only one answer)
   1. Less than 10 (basic commands only, like "sit")
   2. 11 – 30
   3. 31 – 50
   4. More than 50
4. Get dog: How did you get your dog?* (Please mark only one answer)
   1. From the shelter/rescue
   2. I found it on the street
   3. It was born at my home
   4. I bought it from a breeder
   5. I received it as a present
5. Trauma: Has your dog experienced a traumatic event (strong negative experience) which could still have an effect on its behaviour?* (Please mark only one answer)
   1. Yes
   2. No
6. Sex and Neuter status: Gender and neuter status of your dog * (Please mark only one answer)
   1. Intact male
   2. Intact female
   3. Neutered male
   4. Neutered female
7. Dog behaviour changed: Has the behaviour or the personality of your dog changed in the last three months? * (For example, it's activity has decreased/increased; it sleeps less/more at night; it barks less/more; it requires less or more care, has become fearful, etc.) Please mark only one answer)
   1. Yes
   2. No
8. If your dog’s behaviour has changed, how has it changed and in what direction? (For example, need for exercise, barking, sleeping during night or day, house training, showing emotions, relationship with people, personality (e.g. fear, aggression, etc.). Has it increased or decreased?
9. Health problems: What kind of health problems does your dog have? * (You can choose multiple options)
   1. My dog has no problems to the best of my knowledge
   2. Vision problems
   3. Hearing problems
   4. Allergies
   5. Tooth problems, tartar
   6. Joint problems
   7. Dysplasia
   8. Epilepsy
   9. Reproductive problems
   10. Spine problems
   11. Heart failure
   12. Diabetes
   13. Thyroid problems
   14. Cushing’s disease
   15. Trauma/accident complications
   16. Tumor/cancer
   17. Infection
   18. Other
10. Medication: Does your dog regularly take medication? * (Please mark only one answer)
    1. Yes
    2. No
11. Your gender* (Please mark only one answer)
    1. Female
    2. Male
12. Owner age: Your age*
    1. < 20 years
    2. 20 – 29
    3. 30 – 39
    4. 40 – 49
    5. 50 – 59
    6. 60 – 69
    7. 70 – 79
    8. > 79
13. Owner experience: How would you evaluate your experience with dogs?* (You can choose multiple options)
    1. I have not had a dog before this one
    2. I have had dogs before
    3. I am a dog breeder
    4. I am a dog trainer
    5. Dogs are my hobby/profession
    6. I am well-informed on dog-related topics
    7. Other
14. Other dogs in household: How many dogs is your dog living together with?* (Please mark only one answer)
    1. None
    2. 1
    3. 2
    4. 3
    5. 4
    6. 5 or more
15. People in household: How many people is your dog living together with? * (Please mark only one answer)
    1. 1 (myself)
    2. 2
    3. 3
    4. 4
    5. 5 or more
16. Child: Do you have a child/children living with you and your dog? * (Please mark only one answer)
    1. Yes
    2. No
17. Age of dog when arrived: How old was the dog when you got it? * (Please mark only one answer)
    1. < 7 weeks
    2. 7 – 12 weeks
    3. 3 – 12 months
    4. 1 – 2 years
    5. More than 2 years
18. Have any significant changes occurred in the life of the dog? * (You can choose multiple options).
    1. Mating/giving birth
    2. Changes in family structure (for example divorce, birth of children, someone moving away, death in the family..)
    3. Changes in the number of dogs living together (new dog arrived, old dog died)
    4. Moving to a new house
    5. Changing owner
    6. Changes in the time the dog has to spend alone (e.g. due to a new workplace of owner)
    7. The dog has been lost for more than a day
    8. The dog has lived through a traumatic injury/prolonged disease/illness/surgery
    9. None of the options listed above
    10. Other
19. Vitamins: Do you give your dog vitamins or supplements? * (Please mark only one answer)
    1. Regularly (daily)
    2. Often
    3. Rarely
    4. Almost never
    5. Other
20. Where dog is kept: Where do you keep your dog? * Please select which option best describes your dog’s current living situation (where the dog spends the majority of its time). (Please mark only one answer).
    1. In a flat/house with no garden in a typical urban area (few green areas and parks around)
    2. In a flat/house with no garden in a suburban area (many parks and green areas around)
    3. In a flat/house with own fenced garden
    4. In a fenced garden
    5. In a kennel in the yard/garden
    6. In an open area (e.g. in a village or on a farm where the dog is allowed to roam freely)
21. Dog obedience tasks: Out of the tasks below, which can your dog reliably perform? * (You can choose more than one answer).
    1. Sit, lie down
    2. Can be recalled (when off-leash, when interacting with another dog)
    3. Fetch (e.g. dummy, ball)
    4. Stay (at the same place, for at least a minute)
    5. Walk at heel or with a loose leash
    6. “Leave it”/”drop it” (to move away from object/food, or to drop it if already in the mouth)
    7. Watch me (the dog should look up into your face on command)
    8. Barks on command
    9. Waits at road crossings, and does not step off the sidewalk without permission
    10. Can be sent in a specific direction (go forward, or go to objects)
    11. Go to your bed, (dog should go to their bed on command)
    12. Tricks like shake paw, beg, spin, bow, hug, jump etc..
    13. Other
22. Dog training activities: Which of these activities are you currently doing with your dog from the following list?* (You can choose more than one answer).
    1. Short (less than 20 minutes) walks
    2. Long (more than 20 minutes) walks
    3. Hiking, going for trips (more than 1 hour)
    4. Obedience exercises
    5. Running, biking
    6. Trailing, search and rescue
    7. Protection training
    8. Dog shows
    9. Hunting or nose work
    10. Herding
    11. Agility
    12. Frisbee, flyball, or treibball
    13. Swimming
    14. Therapy or service dog
    15. None of the above
    16. Other
23. Time spent alone: How much time does your dog spend alone (without human company) on an average working day? * (Please mark only one answer).
    1. None
    2. 1 – 2 hours
    3. 3 – 8 hours
    4. More than 8 hours

Supplementary Tables/Figures

Table 1: Break down of all the dog breeds present in the sample, including count of the dogs, the percentage of the overall sample, the percentage of the pure breed sample, and the UK Kennel Club breed classification of each breed. The percentages marked in bold are all breeds with a greater than three percentage in the sample population (both overall and in the pure breed sample). (NR = Not recognized by the UK Kennel Club).

| **Breed** | **Count of dogs** | **Percentage of overall sample** | **Percentage of pure breed sample** | **UK Kennel club breed classification** |
| --- | --- | --- | --- | --- |
| Afghan Hound | 1 | 0.08 | 0.13 | Hound |
| Airedale terrier | 3 | 0.25 | 0.38 | Terrier |
| Akita | 9 | 0.75 | 1.14 | Utility |
| Alaskan malamute | 2 | 0.17 | 0.25 | Working |
| American bulldog | 2 | 0.17 | 0.25 | Utility |
| American Staffordshire terrier | 20 | 1.66 | 2.53 | Terrier |
| Dogo Argentino | 2 | 0.17 | 0.25 | Working (NR) |
| Australian kelpie | 1 | 0.08 | 0.13 | Pastoral |
| Australian Shepherd | 2 | 0.17 | 0.25 | Pastoral |
| Basenji | 1 | 0.08 | 0.13 | Hound |
| Basset hound | 2 | 0.17 | 0.25 | Hound |
| Beagle | 25 | 2.07 | **3.16** | Hound |
| Belgian shepherd | 10 | 0.83 | 1.27 | Pastoral |
| Berger Blanc Suisse | 1 | 0.08 | 0.13 | Pastoral (NR) |
| Bernese Mountain dog | 7 | 0.58 | 0.89 | Working |
| Bichon Bolognese | 14 | 1.16 | 1.77 | Toy |
| Bichon Frisé | 1 | 0.08 | 0.13 | Toy |
| Bichon Havanese | 34 | 2.82 | **4.30** | Toy |
| Biewer Terrier (Yorkshire) | 1 | 0.08 | 0.13 | Terrier (NR) |
| Border collie | 34 | 2.82 | **4.30** | Pastoral |
| Borzoi | 2 | 0.17 | 0.25 | Hound |
| Boston terrier | 1 | 0.08 | 0.13 | Utility |
| Bouvier des Flandres | 1 | 0.08 | 0.13 | Working |
| Boxer | 21 | 1.74 | 2.66 | Working |
| Briard | 1 | 0.08 | 0.13 | Pastoral |
| Bull terrier | 8 | 0.66 | 1.01 | Terrier |
| Bull terrier: Miniature | 2 | 0.17 | 0.25 | Terrier |
| Bullmastiff | 1 | 0.08 | 0.13 | Working |
| Cairn terrier | 2 | 0.17 | 0.25 | Terrier |
| Cane corso | 6 | 0.50 | 0.76 | Working (NR) |
| Catalan shepherd dog | 1 | 0.08 | 0.13 | Pastoral |
| Caucasian Shepherd | 1 | 0.08 | 0.13 | Pastoral (NR) |
| Cavalier King Charles | 2 | 0.17 | 0.25 | Toy |
| Central Asian sheepdog | 3 | 0.25 | 0.38 | Pastoral (NR) |
| Chihuahua | 14 | 1.16 | 1.77 | Toy |
| Chow-Chow | 1 | 0.08 | 0.13 | Utility |
| Coton de Tulear | 1 | 0.08 | 0.13 | Toy |
| Dachshund | 35 | 2.90 | **4.43** | Hound |
| Dalmatian | 2 | 0.17 | 0.25 | Utility |
| Dobermann | 5 | 0.41 | 0.63 | Working |
| Dutch Shepherd | 1 | 0.08 | 0.13 | Pastoral (NR) |
| English bulldog | 5 | 0.41 | 0.63 | Utility |
| English cocker spaniel | 18 | 1.49 | 2.28 | Gundog |
| English Setter | 1 | 0.08 | 0.13 | Gundog |
| Fox Terrier | 6 | 0.50 | 0.76 | Terrier |
| French Bulldog | 7 | 0.58 | 0.89 | Utility |
| German shepherd | 35 | 2.90 | **4.43** | Pastoral |
| German Spitz: Miniature | 2 | 0.17 | 0.25 | Utility |
| Golden retriever | 41 | **3.40** | **5.19** | Gundog |
| Gordon setter | 1 | 0.08 | 0.13 | Gundog |
| Great Dane | 6 | 0.50 | 0.76 | Working |
| Hanover Hound (Bloodhound) | 1 | 0.08 | 0.13 | Hound (NR) |
| Hovawart | 1 | 0.08 | 0.13 | Working |
| Hungarian greyhound | 6 | 0.50 | 0.76 | Hound (NR) |
| Vizsla | 58 | **4.81** | **7.34** | Gundog |
| Irish setter | 1 | 0.08 | 0.13 | Gundog |
| Jack Russel Terrier | 4 | 0.33 | 0.51 | Terrier |
| Jagdterrier (Hunting Terrier) | 3 | 0.25 | 0.38 | Terrier (NR) |
| Japanese Chin | 1 | 0.08 | 0.13 | Toy |
| Kangal | 1 | 0.08 | 0.13 | Pastoral |
| Komondor | 1 | 0.08 | 0.13 | Pastoral |
| Kuvasz | 4 | 0.33 | 0.51 | Pastoral |
| Labrador retriever | 59 | **4.89** | **7.47** | Gundog |
| Lagotto Romagnolo | 1 | 0.08 | 0.13 | Gundog |
| Lowchen | 1 | 0.08 | 0.13 | Toy |
| Miniature Pinscher | 9 | 0.75 | 1.14 | Toy |
| Mixed breed | 417 | **34.55** |  |  |
| Moscow Watchdog | 2 | 0.17 | 0.25 | Working (NR) |
| Mudi | 12 | 0.99 | 1.52 | Pastoral (NR) |
| Newfoundland | 1 | 0.08 | 0.13 | Working |
| Old English Sheepdog | 1 | 0.08 | 0.13 | Pastoral |
| Papillon | 2 | 0.17 | 0.25 | Toy |
| Parson Russell Terrier | 4 | 0.33 | 0.51 | Terrier |
| Pekingese | 10 | 0.83 | 1.27 | Toy |
| Perro de Presa Canario | 3 | 0.25 | 0.38 | Working (NR) |
| Pit bull terrier | 3 | 0.25 | 0.38 | Terrier |
| Pointer | 4 | 0.33 | 0.51 | Gundog |
| Poodle | 10 | 0.83 | 1.27 | Utility |
| Poodle: Standard | 1 | 0.08 | 0.13 | Utility |
| Poodle: toy | 7 | 0.58 | 0.89 | Utility |
| Pug | 10 | 0.83 | 1.27 | Toy |
| Puli | 12 | 0.99 | 1.52 | Pastoral |
| Pumi | 3 | 0.25 | 0.38 | Pastoral |
| Pyrenean Mountain Dog | 2 | 0.17 | 0.25 | Pastoral |
| Rottweiler | 7 | 0.58 | 0.89 | Working |
| Rough collie | 6 | 0.50 | 0.76 | Pastoral |
| Schnauzer | 3 | 0.25 | 0.38 | Working |
| Schnauzer: Giant | 5 | 0.41 | 0.63 | Working |
| Schnauzer: Miniature | 10 | 0.83 | 1.27 | Utility |
| Shar Pei | 4 | 0.33 | 0.51 | Utility |
| Shetland sheepdog | 5 | 0.41 | 0.63 | Pastoral |
| Shih Tzu | 17 | 1.41 | 2.15 | Utility |
| Siberian husky | 7 | 0.58 | 0.89 | Working |
| Spaniel | 5 | 0.41 | 0.63 | Gundog |
| Saint Bernard | 1 | 0.08 | 0.13 | Working |
| Staffordshire bull terrier | 2 | 0.17 | 0.25 | Terrier |
| Tibetan spaniel | 5 | 0.41 | 0.63 | Utility |
| Tibetan terrier | 2 | 0.17 | 0.25 | Utility |
| Tosa Inu | 1 | 0.08 | 0.13 | Working (NR) |
| Transylvanian Hound | 2 | 0.17 | 0.25 | Hound (NR) |
| Welsh springer spaniel | 1 | 0.08 | 0.13 | Gundog |
| Welsh terrier | 2 | 0.17 | 0.25 | Terrier |
| West highland white terrier | 24 | 1.99 | **3.04** | Terrier |
| Whippet | 2 | 0.17 | 0.25 | Hound |
| Yorkshire terrier | 36 | 2.98 | **4.56** | Toy |
| Total | 1207 | 100.00 | 100.00 |  |
|  |  |  |  |  |

Table 2: Distribution of the pure breed dogs to the UK Kennel club breed group classifications, including a count of the number of breeds, total number of dogs, and percentage of dogs in each group.

|  |  |  |  |  |  | | |  | |
| --- | --- | --- | --- | --- | --- | --- | --- | --- | --- |
|  | **UK Kennel club Breed Group** | **Count of Breeds** | **Total number of dogs** | **Percentage of pure breed dogs** | | |  | |  |
|  | Gundog | 11 | 190 | 24.05% | |  |  | |  |
|  | Hound | 10 | 77 | 9.75% | |  |  | |  |
|  | Pastoral | 21 | 137 | 17.34% | |  |  | |  |
|  | Terrier | 14 | 84 | 10.63% | |  |  | |  |
|  | Toy | 13 | 135 | 17.09% | |  |  | |  |
|  | Utility | 16 | 85 | 10.76% | |  |  | |  |
|  | Working | 19 | 82 | 10.38% | |  |  | |  |
|  | **Grand Total** | **104** | **790** | **100.00%** | |  |  | |  |
|  |  |  |  |  |  | | |  | |

Figure 1: Distribution patterns for healthy dogs, showing the percentage that remained healthy within one-year age bands. A) mixed breed dogs (n=171), B) purebred dogs (n=305). Unpaired t test: t = 2.404, df = 474, P = 0.017, (Mixed breed: mean = 71.02, SD = 40.34, N = 171, Pure breed: mean = 62.02, SD = 38.55, N = 305).


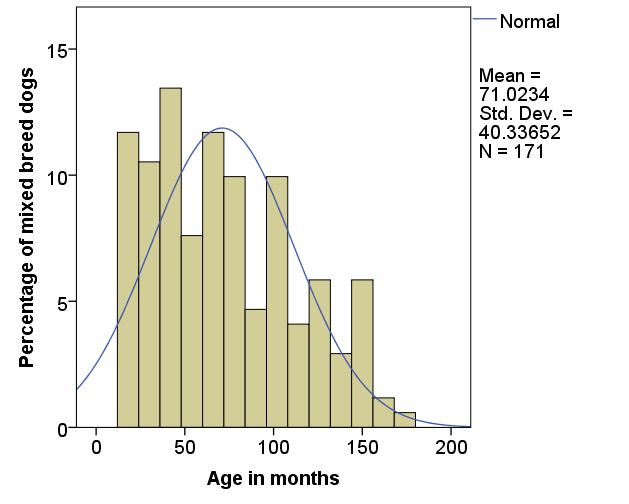


A)


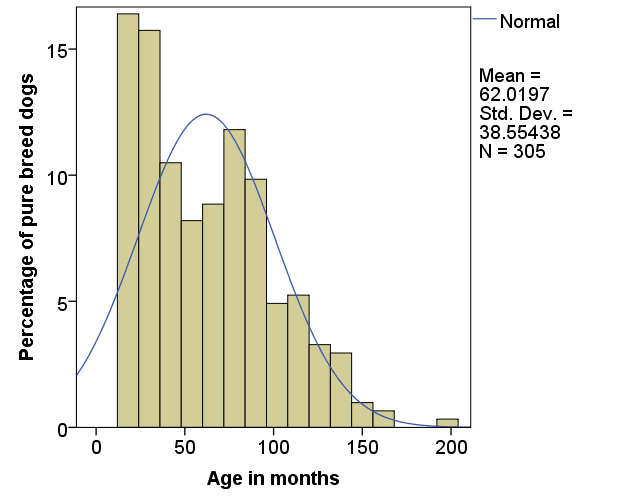


B)

Figure 2: Distribution patterns for unhealthy dogs, showing the percentage that were classified as unhealthy within one-year age bands. A) mixed breed dogs (n=246), B) purebred dogs (n=485). Unpaired t test: t = 2.346, df = 729, P = 0.019, (Mixed breed: mean = 115.91, SD = 49.67, N = 246, Pure breed: mean = 107.27, SD = 45.67, N = 485).


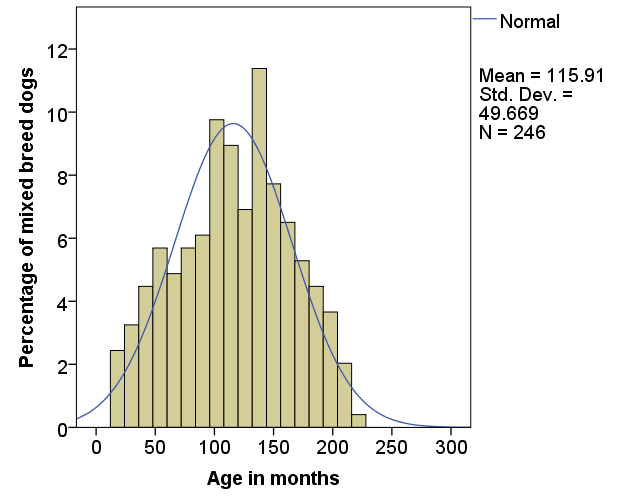


B)

A)


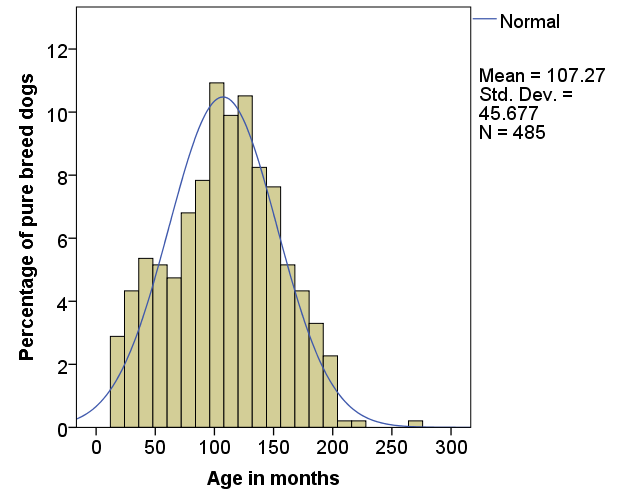

Supplement: Supplementary file 1 [file Table_1.DOCX]
